# Dual-Doped Nickel Sulfide for Electro-Upgrading Polyethylene Terephthalate into Valuable Chemicals and Hydrogen Fuel

**DOI:** 10.1007/s40820-023-01181-8

**Published:** 2023-09-11

**Authors:** Zhijie Chen, Renji Zheng, Teng Bao, Tianyi Ma, Wei Wei, Yansong Shen, Bing-Jie Ni

**Affiliations:** 1 Centre for Technology in Water and Wastewater, School of Civil and Environmental Engineering, University of Technology Sydney, Sydney, NSW 2007 Australia; 2 School of Minerals Processing and Bioengineering, Central South University, Changsha, 410083 People’s Republic of China; 3 School of Biology, Food and Environment Engineering, Hefei University, Hefei, 230601 People’s Republic of China; 4 School of Science, STEM College, RMIT University, Melbourne, VIC 3000 Australia; 5 School of Chemical Engineering, University of New South Wales, Sydney, NSW 2052 Australia

**Keywords:** Hydrogen energy, Electro-upcycling, Structural reconstruction, Organic waste upcycling, d Band centre

## Abstract

Electro-upcycling of plastic waste into value-added chemicals/fuels is an attractive and sustainable way for plastic waste management. Recently, electrocatalytically converting polyethylene terephthalate (PET) into formate and hydrogen has aroused great interest, while developing low-cost catalysts with high efficiency and selectivity for the central ethylene glycol (PET monomer) oxidation reaction (EGOR) remains a challenge. Herein, a high-performance nickel sulfide catalyst for plastic waste electro-upcycling is designed by a cobalt and chloride co-doping strategy. Benefiting from the interconnected ultrathin nanosheet architecture, dual dopants induced up-shifting d band centre and facilitated in situ structural reconstruction, the Co and Cl co-doped Ni_3_S_2_ (Co, Cl-NiS) outperforms the single-doped and undoped analogues for EGOR. The self-evolved sulfide@oxyhydroxide heterostructure catalyzes EG-to-formate conversion with high Faradic efficiency (> 92%) and selectivity (> 91%) at high current densities (> 400 mA cm^−2^). Besides producing formate, the bifunctional Co, Cl-NiS-assisted PET hydrolysate electrolyzer can achieve a high hydrogen production rate of 50.26 mmol h^−1^ in 2 M KOH, at 1.7 V. This study not only demonstrates a dual-doping strategy to engineer cost-effective bifunctional catalysts for electrochemical conversion processes, but also provides a green and sustainable way for plastic waste upcycling and simultaneous energy-saving hydrogen production.

**Supplementary Information:**

The online version contains supplementary material available at 10.1007/s40820-023-01181-8.

## Highlights

- Co and Cl co-doped NiS is an efficient bifunctional electrocatalyst for converting plastic waste into formate and hydrogen with high efficiency and selectivity
- Dopants regulate the electronic property and accelerate structural reconstruction of NiS for the core ethylene glycol (PET monomer) oxidation reaction
- PET hydrolysate electrolysis can produce hydrogen gas at an average rate of 50.26 mmol h^−1^ at 1.7 V

**Supplementary Information:**

The online version contains supplementary material available at 10.1007/s40820-023-01181-8.

## Graphical abstract

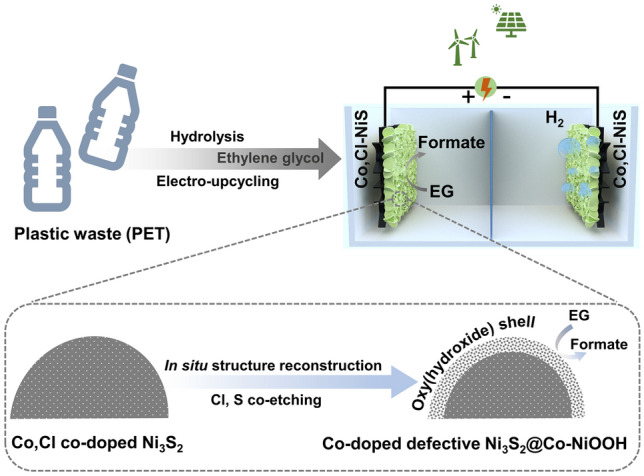

## Introduction

Polyethylene terephthalate (PET) is an important engineering polymer, and it is one of the most widely used plastics in the world [1]. Currently, ~ 70 million tons of PET are produced annually in the world, while most of them are landfilled or incinerated directly, leading to severe environmental pollution [2–4]. Handling PET waste is thus an important mission, and thermal recycling methods are the most widely used to convert PET into value-added products [5, 6]. At elevated temperatures, the thermochemical approaches can convert PET into carbon materials, chemicals, and fuels [7–10]. However, thermal conversion generally leads to high carbon emissions, low product selectivity, and needs complicated devices [11, 12], which makes it unsustainable for the clean recycling of PET waste [13].

Alternatively, photochemical, photoelectrochemical, and electrochemical upcycling of PET has been proposed recently as mild methods to obtain value-added chemicals/fuels with high purity [14–18]. Among these route, electro-upcycling distinguishes itself for its high efficiency and attracts growing interest [19]. In a typical electro-upcycling process, PET is first hydrolyzed into ethylene glycol (EG) and terephthalate (TPA) monomers, and EG in the hydrolysate can be further electrooxidized into glycolic acid, formate, carbonate, etc. [20, 21]. The products of EG oxidation reaction (EGOR) are highly related to the applied electrocatalysts, and previous studies have shown that several low-cost transitional metal (TM) -based catalysts (e.g., CuO [22], NiCo_2_O_4_ [23], Co-Ni_3_N [24], CuCoO_4_ [11], CoNi_0.25_P [25], Co-Ni_2_P [26]) can catalyze EGOR for selective formate production. Nevertheless, the development of highly electroactive, selective, earth-abundant, and stable EGOR electrocatalysts remains a big challenge, and current work mainly focuses on TM oxides, phosphides, and nitrides. TM sulfides, with high earth abundance, good electronic/chemical properties, and environmental friendliness [27, 28], have exhibited good electrochemical performance for diverse reactions, such as urea oxidation reaction, carbon dioxide reduction, batteries, oxygen evolution reaction (OER), and hydrogen evolution reaction (HER) [29–36]. However, the design of efficient TM sulfides for EGOR is largely unexplored. To enhance the catalytic performance of TM sulfides, strategies like elemental doping is widely used [37, 38]. The introduction of dopants can regulate TM sulfides’ electronic structure and/or conductivity, thus enhancing the catalytic performance [39]. Compared to conventional single cation or anion doping, cation–anion dual doping emerges as a powerful method to optimize TM sulfides’ multiple physicochemical properties simultaneously [40]. Additionally, previous studies found that TM-based catalysts would undergo structural reconstruction during the EGOR process, and the in situ formed metal (oxy)hydroxides played a critical role in determining the catalytic performance [25, 41–44]. In this regard, developing high-performance TM sulfides with controllable structural reconstruction by efficient doping strategies would largely advance the EGOR process and accelerate the recycling of PET. Moreover, uncovering the conversion mechanism of EG over TM sulfides and revealing the structure–activity relationship is of great significance for designing next-generation catalysts for plastic utilization, which remains untouched.

On the other hand, coupling a thermodynamically favourable oxidation reaction with HER holds the promise for energy-saving hydrogen production compared with conventional water electrolysis [45–47]. Recent reports have proved that EGOR with a low theoretical oxidation potential (0.57 V vs. reversible hydrogen electrode [RHE]) is a great half-reaction to replace OER (theoretical oxidation potential of 1.23 V vs. RHE) [48]. To this end, exploring high-performance TM sulfide catalysts with high electroactivity, selectivity, and stability for EGOR is urgent for enhancing the reaction efficiency and cutting the production cost for chemicals and green hydrogen fuel.

Here, we develop an anion-cation co-doping strategy to improve the catalytic performance of nickel sulfide for converting real PET waste into hydrogen and formate. The Co and Cl co-doped nickel sulfide (Co, Cl-NiS) prepared by a one-step hydrothermal route shows better performance toward EGOR than the single-doped and undoped counterparts. Co, Cl-NiS only needs 1.346 V versus reversible hydrogen electrode (RHE) to achieve 100 mA cm^−2^ for EGOR, and it can realize high efficiency and selectivity for EG-to-formate conversion at high current densities. Further analyses indicate the excellent catalytic properties of Co, Cl-NiS stem from the ultrathin nanosheet structure, and the dopants regulated electronic structure and facilitated in situ structure reconstruction. For the real PET waste hydrolysate electrolysis in a membrane-electrode assembly (MEA) electrolyzer, the bifunctional Co, Cl-NiS can attain a high H_2_ production rate. Generally, nickel sulfide-based low-cost catalysts-mediated plastic upcycling technique would help to address current pressing plastic pollution and innovate further high-performance catalyst development.

## Experimental Section

### Synthesis of Catalysts

All nickel sulfide catalysts were fabricated by a facile hydrothermal method. For the preparation of Co, Cl co-doped nickel sulfide catalyst (denoted as Co, Cl-NiS), 0.5 mmol thiourea, 0.01 mmol NaCl, and 0.01 mmol Co(NO_3_)_2_·6H_2_O were first added into a beaker containing 10 mL deionized (DI) water. The mixture was then stirred for half an hour at 400 rmp; it was then poured into a Teflon-lined stainless steel autoclave (25 mL). Then, a piece of acidic treated nickel foam (NF, with a thickness of 2 mm) was totally immersed in the homogeneous solution. The reactor was put in a conventional oven and treated at 120 °C (12 h). After cooling naturally, the as-prepared Co, Cl-NiS material was cleaned and dried. At last, the Co, Cl-NiS catalyst was obtained. The undoped nickel sulfide catalyst (NiS), Co-doped nickel sulfide catalyst (Co-NiS), and Cl-doped nickel sulfide catalyst (Cl-NiS) were also synthesized via a similar method described above for comparison.

### Electro-Upcycling of Real Plastics

The electro-upcycling of PET waste contains two steps, including the alkaline hydrolysis and the following PET hydrolysate electrolysis.

*Alkaline hydrolysis of PET bottles*. The collected PET waste was first cleaned and cut into tiny pieces (≤ 1 mm) for the hydrolysis treatment. Per 10 g of dried plastic particles were dispersed in 100 mL of 2 M KOH solution in a flask. Then, the flask was sealed with a rubber stopper and heated on a hotplate at 80 °C with stirring (800 rpm) for 12 h. Afterward, the hydrolysate was collected for electrolysis.

*PET hydrolysate electrolysis*. The PET hydrolysate electrolysis and conventional water electrolysis were conducted with a home-made MEA electrolyzer for 24 h. Each side of the MEA electrolyzer has a volume of ~ 20 mL. The as-prepared self-supported Co, Cl-NiS (mass loading of ~ 0.9 mg cm^−2^) with a size of 2.5 × 2.5 cm^2^ was directly used as the anode and the cathode. An anion exchange membrane (AEM, Sustainion X37-50, Dioxide Materials) with a thickness of 50 μm was used between the cathode and anode sections. The NF-based electrode itself works as a diffusion layer, and the gasket thickness was adjusted according to the NF thickness employed. The measurements were performed at room temperature, and 2 M KOH aqueous solution and 2 M KOH/20 g L^−1^ PET hydrolysate were separately added into the cathode and anode sides with a flow rate of 0.2 mL min^−1^ with a peristaltic pump. The gas and liquid products were collected and analyzed by gas chromatography (GC) and nuclear magnetic resonance (NMR) spectroscopy respectively. For the Co, Cl-NiS assisted conventional water electrolysis tests, 2 M KOH solution was used as the electrolyte in both cathode and anode sides, with a flow rate of 0.2 mL min^−1^.

Details about the chemicals, electrochemical tests, catalyst characterizations, and computational details are presented in the Supporting Information.

## Results and Discussion

### Catalyst Characterization

**Fig. 1:**
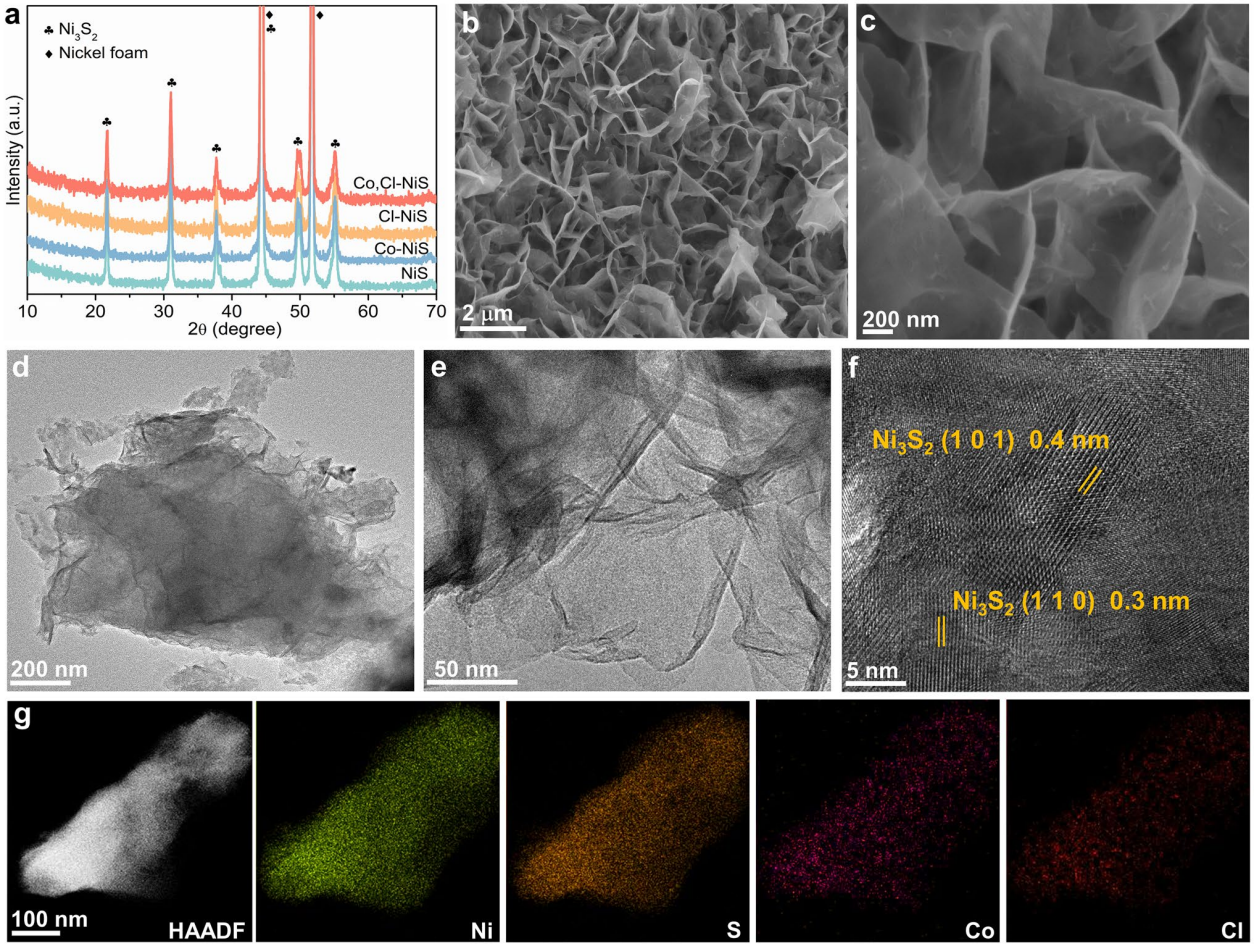
**a** XRD patterns of catalysts. **b**, **c** SEM images of Co, Cl-NiS. **d**, **e** TEM images of Co, Cl-NiS. **f** HRTEM images of Co, Cl-NiS. **g** HAADF-STEM and corresponding elemental mapping images of Co, Cl-NiS

All nickel sulfide catalysts were developed by a one-step hydrothermal process. As shown in the X-ray diffraction (XRD) patterns (Fig. 1a), all characteristic diffraction peaks of the bare nickel sulfide (NiS) catalyst can be ascribed to the Ni_3_S_2_ phase (JCPDS no. 44-1418), except two strong peaks of the NF substrate (Ni, JCPDS no. 04-0850). Compared to the undoped NiS sample, the XRD patterns of Co- or Cl- doped NiS and the Co, Cl co-doped NiS catalysts show no new peaks, suggesting the doping of Co and/or Cl does not alter the main Ni_3_S_2_ phase. The morphology structure of the as-obtained Co, Cl-NiS sample was checked with scanning electron microscopy (SEM) and transition electron microscopy (TEM). In Fig. 1b, c, abundant vertical nanosheets evenly grown on the three-dimensional (3D) NF can be observed, with an average lateral length of about 2 microns and thickness of 20 nm. These interconnected ultrathin nanosheets would provide rich active sites for electrochemical reactions [49]. Furthermore, TEM images support the generation of sheet-like nanostructure of Co, Cl-NiS (Fig. 1d, e). In addition, three distinct lattice fringes with interplanar distances of 0.3, and 0.4 nm in Fig. 1f can be indexed to the (110) and (101) planes of Ni_3_S_2_, respectively. Furthermore, high-angle annular dark-field scanning transmission electron microscopy (HAADF-STEM) and corresponding elemental mapping images in Fig. 1g show an even spatial distribution of S (yellow), Cl (red), Co (purple), and Ni (green) elements in the Co, Cl-NiS sample, indicating the successful introduction of Co and Cl elements.

**Fig. 2:**
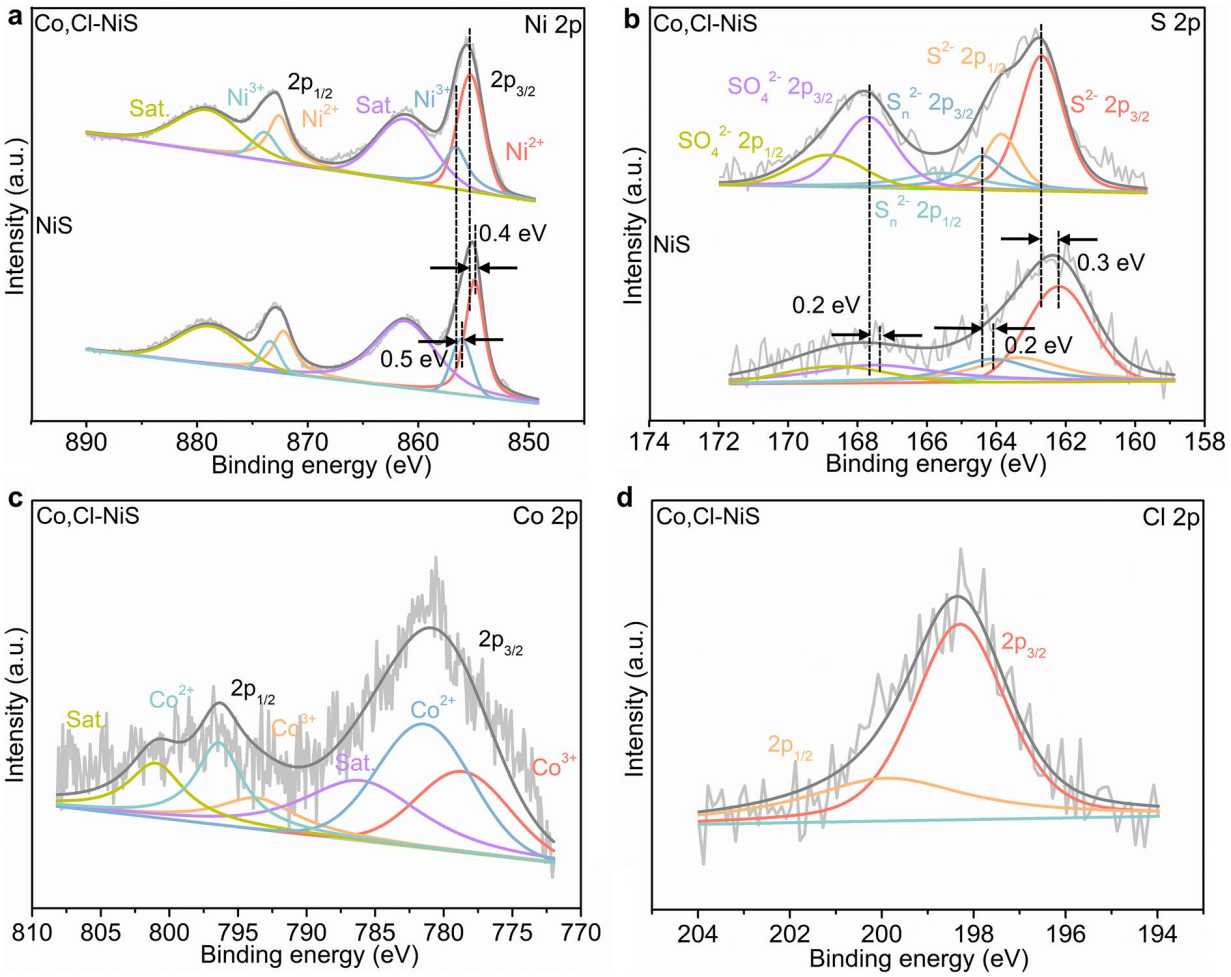
a Ni 2*p*, **b** S 2*p*, **c** Co 2*p*, and **d** Cl 2*p* XPS spectra of Co, Cl-NiS and NiS

The surface compositions and chemical states of Co, Cl-NiS and undoped NiS were investigated by X-ray photoelectron spectroscopy (XPS). Figure S1 suggests the co-occurrence of S, Ni, Co, and Cl elements in Co, Cl-NiS and the co-presence of Ni and S elements in undoped NiS. In the Ni 2*p* spectrum of undoped NiS, two distinct peaks at 854.9 and 872.2 eV can be ascribed to 2*p*_3/2_ and 2*p*_1/2_ states of Ni^2+^, respectively [50, 51] (Fig. 2a). Peaks at 856.1 and 873.4 eV are assigned to Ni^3+^ 2*p*_3/2_ and Ni^3+^ 2*p*_1/2_ respectively, with two satellite peaks at 861.2 and 878.5 eV [52]. Compared to bare NiS, the binding energies of Ni^2+^ 2*p*_3/2_ (855.3 eV) and Ni^3+^ 2*p*_3/2_ (856.3 eV) in Co, Cl-NiS show a positive shift, indicating a reduced electron density around Ni atoms after the Co, Cl co-doping. The reason should be that Cl dopant with a high electronegativity could extract electrons from surrounding Ni atoms and thereby decrease the electronic density around Ni atoms [53, 54]. Of note, the Ni sites of Co, Cl-NiS show a reduced electron density and a higher Ni^3+^/Ni^2+^ ratio than NiS (0.47 of Co, Cl-NiS vs. 0.39 of NiS). In Fig. 2b, the S 2*p* spectrum of bare NiS is fitted with six peaks. Two peaks at 162.2 and 163.4 eV correspond to 2*p*_3/2_ and 2*p*_1/2_ states of S^2–^, and two peaks located at 164.1 and 165.3 eV are assigned to S 2*p*_3/2_ and S 2*p*_1/2_ of S_n_^2–^, respectively. Two other peaks at the higher energy region are referred to 2*p*_3/2_ (167.5 eV) and 2*p*_1/2_ (168.6 eV) states of SO_4_^2–^ [55, 56]. All peaks of S^2–^, S_n_^2–^, and SO_4_^2–^ in the S 2*p* spectrum of Co, Cl-NiS show positive shifts relative to those in the spectrum of undoped NiS, implying a lowered electron density around S atoms with Co and Cl co-doping. The reason should be that the Cl dopant which has the strongest electronegativity than Ni/Co and S can attract electrons from Ni/Co and S, and thus reduce the electron density on the S sites [57]. The Co 2*p* spectrum of Co, Cl-NiS in Fig. 2c contains six peaks, including 2*p*_3/2_ (778.6 eV) and 2*p*_1/2_ (793.6 eV) states of Co^3+^, 2*p*_3/2_ (781.4 eV) and 2*p*_1/2_ (796.4 eV) states of Co^2+^, and two satellite peaks at 786 and 801 eV [58]. The presence of high-valence Ni^3+^, Co^3+^, and SO_4_^2–^ is mainly due to the superficial oxidation of catalysts in the air [59, 60]. Moreover, in the Cl spectrum of Co, Cl-NiS (Fig. 2d), two characteristic peaks at 198.3 and 199.9 eV can be found, which are indexed as Cl 2*p*_3/2_ and Cl 2*p*_1/2_, respectively [61]. Spectra of Co and Cl validate that both Co and Cl elements have been incorporated into NiS successfully, and the Co, Cl co-doping significantly regulates the electronic property of NiS.

### EGOR Performance of Catalysts

**Fig. 3:**
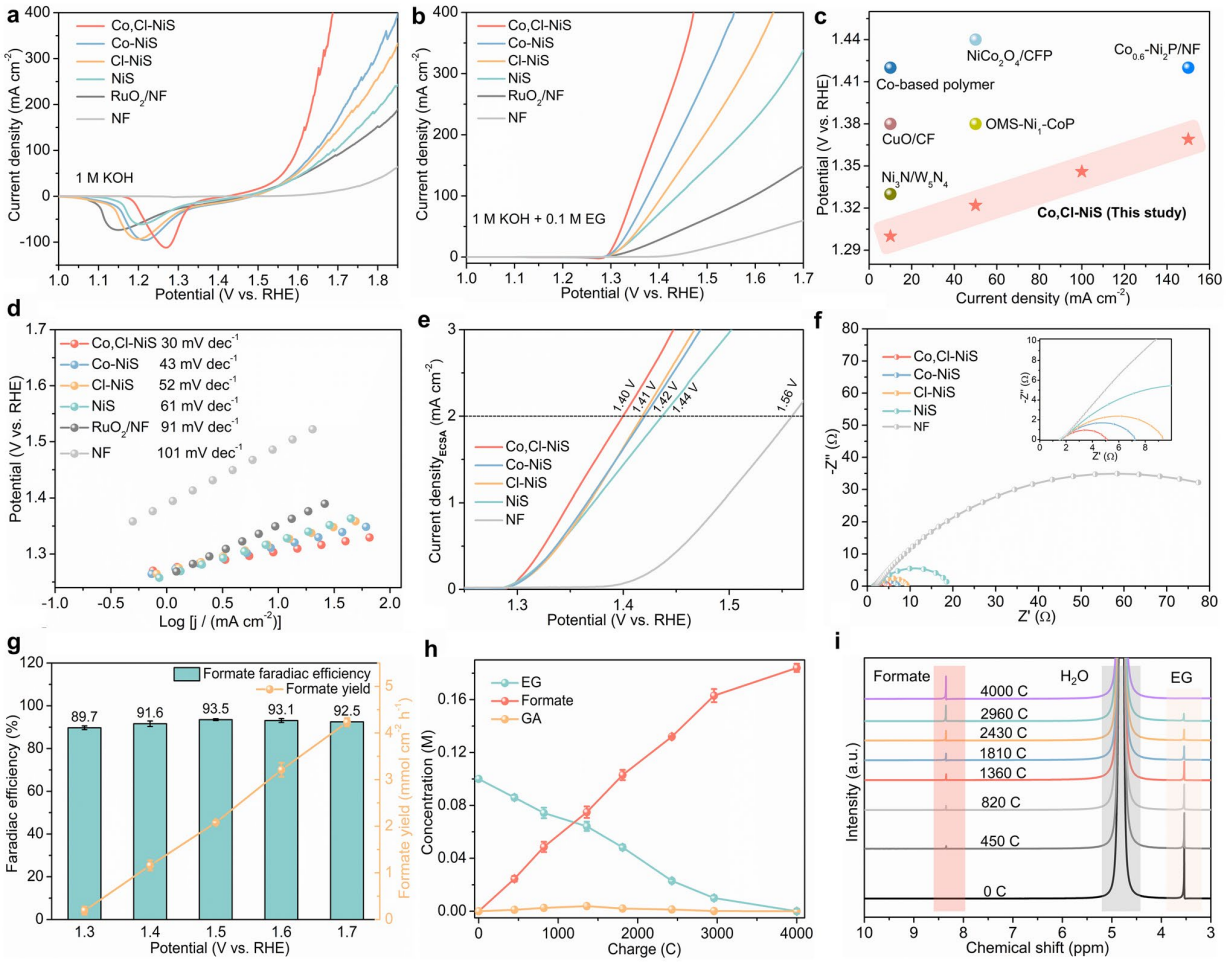
a OER LSV curves of electrocatalysts. **b** EGOR LSV curves of catalysts. **c** Comparison of Co, Cl-NiS with state-of-the-art noble metal-free electrocatalysts for EGOR. **d** Corresponding Tafel plots for EGOR. **e** ECSA normalized EGOR LSV curves. **f** EIS Nyquist plots, insert shows an enlarged part of Nyquist plots. **g** Faradic efficiency and yield for formate production of Co, Cl-NiS at different potentials. **h** Concentrations of EG, GA, and formate in the charge-dependent EGOR process. **i** ^1^H NMR spectra of products during EGOR process

The electrochemical performance of catalysts towards OER and EGOR was investigated with a H-type cell. Aside from prepared nickel sulfide catalysts, the RuO_2_ electrocatalyst, and pure NF were also studied. The OER performance was tested in 1 M KOH electrolyte. As shown in linear sweep voltammetry (LSV) curves (Fig. 3a), the nickel sulfide-based catalysts exhibit better OER performance than the RuO_2_ catalyst and NF, and the Co and Cl co-doped sample outperforms the Co or Cl single-doped and undoped counterparts. At an overpotential (*η*) of 400 mV, Co, Cl-NiS attains a higher current density (182 mA cm^−2^) towards OER than Co-NiS (85 mA cm^−2^), Cl-NiS (76 mA cm^−2^), NiS (60 mA cm^−2^), NF (4.5 mA cm^−2^), and the benchmark RuO_2_ catalyst (55 mA cm^−2^). Apparently, the current density of the NF support is quite low, indicating that the electrocatalytic activity of as-prepared electrodes mainly comes from the nickel sulfide phase. Of note, the *η* at 10 mA cm^−2^ (*η*_10_) of Co, Cl-NiS (241 mV) is lower than state-of-the-art TM-based OER catalysts, such as FeCoNiMo high-entropy alloy (250 mV) [62] and Ti-CoS_x_ (249 mV) [63], and Table S1 presents more comparisons.

EGOR performance of catalysts was measured in 1 M KOH with the addition of 0.1 M EG. LSV curves in Fig. 3b suggest that the EGOR activity trend of electrocatalysts follows the OER activity. Compared with undoped, single Co or Cl doped nickel sulfide catalysts, Co, Cl-NiS exhibit better EGOR activities. Potentials at 100 mA cm^−2^ for Co, Cl-NiS, Co-NiS, Cl-NiS, NiS and the RuO_2_ catalyst are about 1.34, 1.38, 1.41, 1.44, and 1.60 V vs. RHE respectively. The doping levels of Cl and Co were optimized based on the catalytic performance, and detailed information is shown in Fig. S2. The best activity is obtained when the amounts of Cl^−^ and Co^2+^ precursors are both 0.01 mol. Compared to OER, NiS-based electrocatalysts achieve a higher current density for EGOR at a given potential. Especially, a large potential difference of 220 mV is observed for Co, Cl-NiS for EGOR and OER, at 300 mA cm^−2^ (Fig. S3). In this case, the OER may compete with EGOR at high potentials and reduce the reaction efficiency of EGOR. Additionally, Co, Cl-NiS exhibits better EGOR activities than state-of-the-art transition metal-based catalysts (Fig. 3c and Table S2), suggesting the huge potential of TM sulfides for the EGOR application. The electrochemical kinetics for EGOR of all tested electrocatalysts were further probed through Tafel plots (Fig. 3d). Co, Cl-NiS showcases a low Tafel slope of 30 mV dec^−1^, better than NF (101 mV dec^−1^), the RuO_2_ catalyst (91 mV dec^−1^), NiS (61 mV dec^−1^), Cl-NiS (52 mV dec^−1^), and Co-NiS (43 mV dec^−1^). In this context, Co, Cl-NiS has optimal thermodynamic and kinetic EGOR performance.

Electrochemically active surface area (ECSA) is a critical electrochemical property of catalysts, which can help to quantify the number of catalysts’ active sites. Based on the electrochemical double-layer capacitances (C_dl_) results (Fig. S4-S5), Co, Cl-NiS has a larger C_dl_ value (4.31 mF cm^−2^) over Co-NiS (3.37 mF cm^−2^), Cl-NiS (2.25 mF cm^−2^), NiS (1.97 mF cm^−2^), as well as NF (0.54 mF cm^−2^). With a larger ECSA (107 cm^2^), Co, Cl-NiS thus can offer more catalytically active area for electrochemical reactions than its counterparts. Furthermore, the EGOR LSV curves were normalized with ECSA to exclude the impact of ECSA on understanding the intrinsic catalytic activity. Co, Cl-NiS only needs a potential of 1.40 V vs. RHE to gain a *j*_ECSA_ of 2 mA cm^−2^ (Fig. 3e), lower than Co-NiS (1.41 V vs. RHE), Cl-NiS (1.42 V vs. RHE), NiS (1.44 V vs. RHE), and NF (1.56 V vs. RHE). These features (large ECSA, high intrinsic activity) of Co, Cl-NiS contribute to its excellent EGOR performance. Moreover, the Co or Cl single doping can upgrade the ECSA and intrinsic activity of bare nickel sulfide, which is further enhanced by the co-presence of Co and Cl dopants.

The charge transfer properties (charge-transfer resistance (R_ct_)) of catalysts during the EGOR process were studied with electrochemical impedance spectroscopy (EIS). shows The Nyquist plots in Fig. 3f were fitted with an equivalent circuit model (Fig. S6), and the results are displayed as Table S3. Co, Cl-NiS has a largely smaller R_ct_ (3.7 Ω) than NF (123 Ω), NiS (19.5 Ω), Cl-NiS (8.4 Ω), and Co-NiS (6.1 Ω). Accordingly, the Co, Cl-NiS catalyst endows an efficient charge transfer feature is during EGOR, when using. Both Co and Cl doping can enhance the charge transfer kinetics of nickel sulfide, which is further improved by the Co and Cl co-doping. Besides high activities, Co, Cl-NiS also has high stability for EGOR. Figure S7 illustrates that the catalytic activity can maintain at about 86% in the 12 h chronoamperometry (CA) test. Additionally, the LSV curve of Co, Cl-NiS after the CA test in the pristine 1 M KOH + 0.1 M EG solution is almost the same as the one before the CA test. Hence, the main reason for the decreased current density should be the reduced EG concentration [42].

To figure out the conversion process of EG, ^1^H and ^13^C nuclear magnetic resonance (NMR) spectroscopy was used to confirm EG electro-oxidation products, qualitatively and quantitatively. As presented in Fig. 3g, the main product is formate, with ~ 90% of faradic efficiency (FE) in the potential region of 1.3–1.7 V versus RHE, suggesting a high selectivity of EG conversion over Co, Cl-NiS. The highest FE (93.5%) and selectivity (93.9%, Fig. S8) of formate was attained at 1.5 V vs. RHE, and further higher potentials lead to slightly reduced FE. This should be the deep oxidation of formate or water oxidation at high potentials [22]. The formate yield was also calculated, which roughly shows a positive correlation with the applied potential. A formate yield of 4.26 mmol cm^−2^ h^−1^ is obtained at 1.7 V vs. RHE. To gain insights into the dynamic conversion of EG during the oxidation process, input charge-dependent NMR spectra were recorded. As the electro-oxidation proceeds, the concentration of EG decreases gradually and the concentration of formate shows a continuous increase (Fig. 3h). With an input charge of 4000 C, all EG has been oxidized and only a low proportion of glycolate (GA) is formed during the oxidation process, and formate is obtained with good FE (93.5%) and high carbon balance (> 94%, Fig. S9). The EG conversion process is further evidenced by the ^1^H and ^13^C NMR spectra (Figs. 3i and S10). In the ^1^H NMR spectra, the peak of EG gradually weakens until disappears, and the peak of formate becomes stronger, with the continuous electro-oxidation process. An enlarged part of the ^1^H NMR spectra in Fig. S11 shows the evolution of GA. It can be seen that a small peak in the chemical shift region of 3.80–3.85 ppm which can be indexed to GA generates first and then disappears. Therefore, a little amount of GA was generated from EG oxidation, and it was further converted to other chemicals as the oxidation process proceeded. The ^13^C NMR spectra of the post-EGOR electrolyte also suggest the production of formate and a small amount of carbonate. Combining the ^1^H NMR and ^13^C spectra, it can be concluded that the GA intermediate converts into formate and carbonate. Based on the NMR results and previous studies [11, 15, 25, 64], a possible pathway of the Co, Cl-NiS-mediated EGOR is suggested. First, EG is oxidized to glycolic aldehyde which is subsequently instantly converted into GA and glyoxal. Second, the cleavage of C–C bonds in glyoxal generates formate, the cleavage of C–C bonds in GA forms carbonate and formate. Notably, only a little amount of GA and carbonate species were detected during the oxidation of EG, implying that GA is a minor reaction intermediate in the EG-to-formate conversion process (Fig. S12).

### Origin of the Excellent EGOR Performance of Co, Cl-NiS

**Fig. 4:**
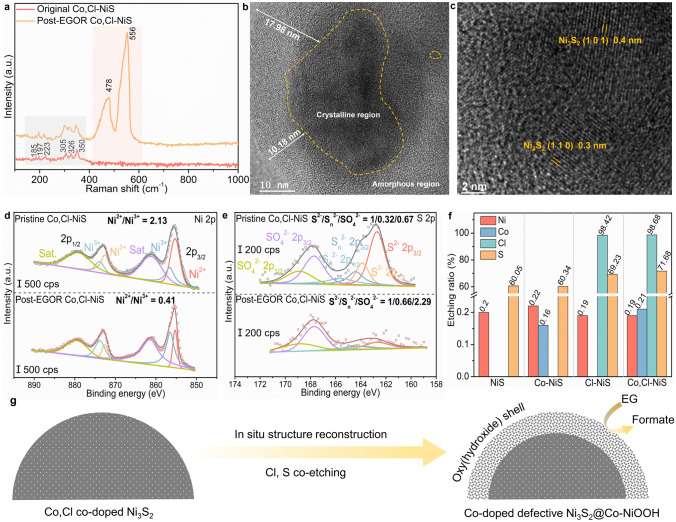
**a** Raman spectra of Co, Cl-NiS before and after EGOR process. **b** TEM and **c** HRTEM images of Post-EGOR Co, Cl-NiS. **d** Ni 2p and **e** S 2p XPS spectra of Co, Cl-NiS before and after EGOR process. **f** Ion concentrations in electrolytes of different catalysts after EGOR process. **g** Illustration of the self-reconstruction process of Co, Cl-NiS

Aside from the interconnected ultrathin nanosheets structure of Co, Cl-NiS that can provide rich electroactive sites and promote charge/mass transfer, post-EGOR characterizations were thoroughly performed with spectroscopic, microscopic, and analytical tools to study the structure-performance correlation of Co, Cl-NiS. The Raman spectra were first recorded, and the results are shown in Fig. 4a. For the as-prepared Co, Cl-NiS, the Raman shifts at 185, 197, 223, 305, 326 and 350 cm^−1^ are in line with the vibrations of Ni_3_S_2_ [65, 66]. Maintaining the characteristic peak of Ni_3_S_2_, post-EGOR Co, Cl-NiS shows two new strong peaks at ~ 478 and ~ 556 cm^−1^ which are indexed to the Ni–O bending and stretching vibrations of γ-NiOOH phase [67, 68]. Accordingly, the Co, Cl-NiS has undergone structure reconstruction during the EGOR process, and the co-existing Ni_3_S_2_ and NiOOH phases account for the high EGOR activity. The structure evolution is further confirmed by the TEM images. In Fig. 4b, the regions with clear lattice fringes are surrounded by dense amorphous NiOOH layers with depth of over 10 nm. The HRTEM images in Fig. 4c indicate that the lattice fringes are ascribed to Ni_3_S_2_, in line with the Raman spectra. Therefore, metal oxyhydroxide (Co-doped NiOOH) is generated on sulfide surface under the EGOR condition.

XPS spectra of post-EGOR Co, Cl-NiS were also collected to analyze the variations in surface chemical properties. In the high-resolution Ni 2*p* spectra, the Ni^2+^/Ni^3+^ ratio decreases from 2.13 (pristine Co, Cl-NiS) to 0.41 (post-EGOR Co, Cl-NiS) (Fig. 4d), suggesting the dominant Ni species in the post-EGOR Co, Cl-NiS are the high-valence Ni^3+^. Similarly, the Co 2*p* spectrum of the post-EGOR Co, Cl-NiS in Fig. S13a also exhibits a higher proportion of high-valence Co^3+^, with the Co^2+^/Co^3+^ ratio reduced from 1.55 (pristine Co, Cl-NiS) to 0.53 (post-EGOR Co, Cl-NiS). These stable high-valence metal species can contribute to high catalytic performance towards EGOR. The oxidation of metal species is accompanied by the oxidation of sulfur. In Fig. 4e, the S^2−^/S_n_^2−^/SO_4_^2−^ ratio of pristine Co, Cl-NiS is 1/0.32/0.67, which is changed to 1/0.66/2.29 of the post-EGOR sample. Aside from the growth in the high-valence S species, it is obvious that the content of S element is significantly decreased. Also, the XPS spectra of Cl shows that the signal of Cl element is almost not detected in the post-EGOR Co, Cl-NiS (Fig. S13b). The significant reduction of S and Cl elements in the post-EGOR Co, Cl-NiS is due to the in situ electrochemical etching during the electro-oxidation process. In the O 1s spectra (Fig. S13c), the peak belongs to lattice oxygen species of post-EGOR Co, Cl-NiS (530.4 eV) increase considerably than the pristine catalyst, further verifying the generation of metal oxyhydroxides.

Additionally, inductively coupled plasma mass spectrometry (ICP-MS) was employed to investigate the chemical composition change of catalysts during EGOR. In Fig. 4f, the electrochemical etching ratios of metals are quite low (~ 0.2%), suggesting the high stability of the catalyst framework. However, both S and Cl show high etching ratios. The Co, Cl-NiS shows a higher S etching ratio of 71.68% than Cl-NiS (69.23%), Co-NiS (60.34%), and NiS (60.05%). It can be seen that the Cl dopant has an obvious effect on facilitating the dissolution of S during the EGOR process, which may be due to the doping-induced more high-valence S species in the Co, Cl-NiS. In addition, the dissolved Cl amounts of Cl-NiS and Co, Cl-NiS are 98.42% and 98.68% respectively, which implies the complete etching of Cl elements. The high electrochemical dissolution of Cl also has been suggested in previous studies on OER [69, 70]. From a water security point of view, the Cl ions with potential toxic effects in the post-reaction solution can be removed by capacitive deionization [71]. The high etching ratio of the Co, Cl-NiS is also evidenced by the elemental mapping images in Fig. S14. The prominent co-etching of sacrificial Cl dopants contributes to rich S-site vacancies and facilitates the structure reconstruction for the formation of electroactive Co-doped NiOOH phase on the surface of sulfide nanosheets (Fig. 4g).

**Fig. 5:**
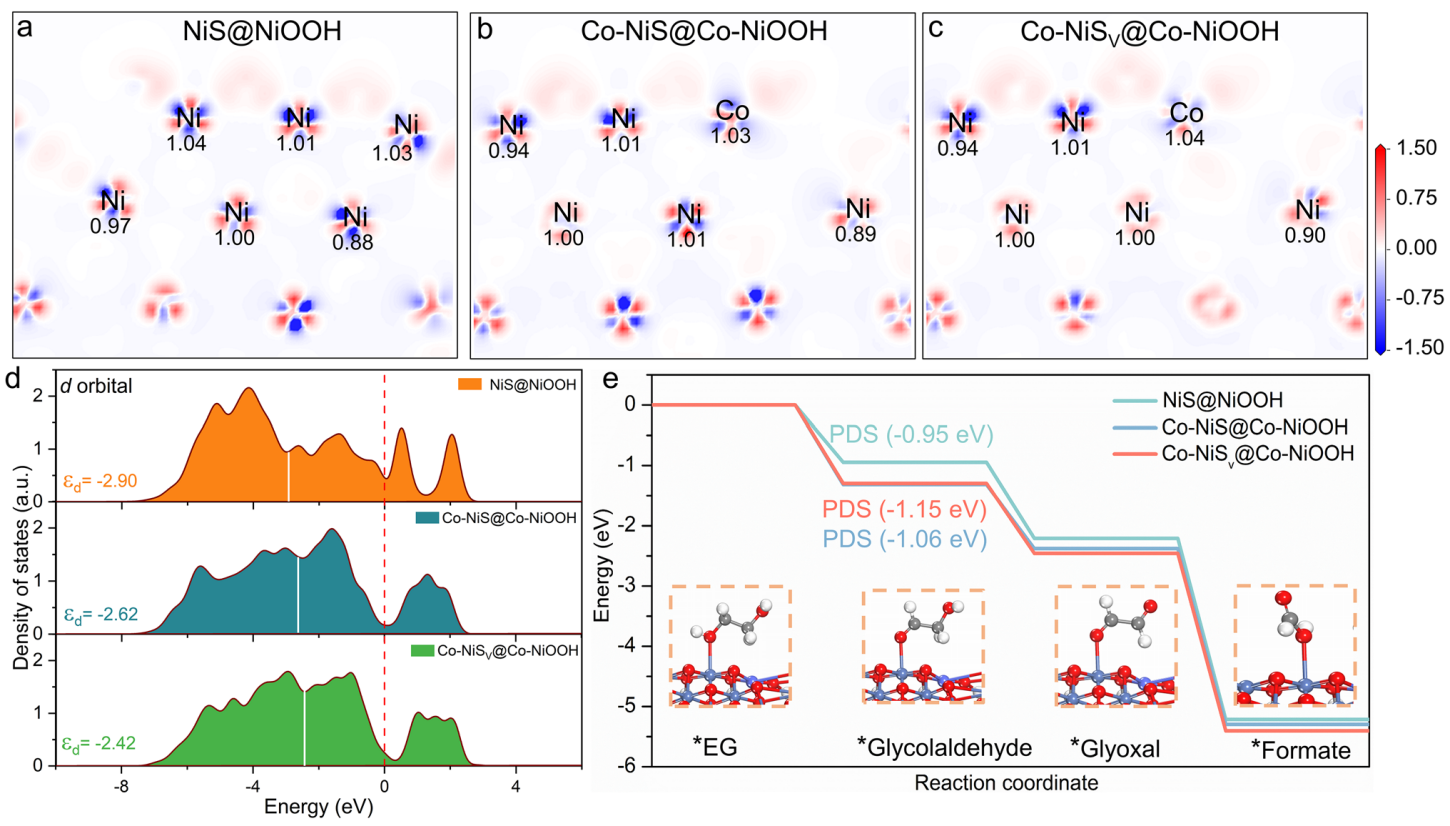
a–c Electron density differences of NiS@NiOOH, Co-NiS@Co-NiOOH, and Co-NiS_v_@Co-NiOOH (red and blue colours illustrate electron accumulation and depletion, respectively). **d** DOS of NiS@NiOOH, Co-NiS@Co-NiOOH, and Co-NiS_v_@Co-NiOOH. **e** Gibbs free energy diagrams for EGOR over the surfaces of NiS@NiOOH, Co-NiS@Co-NiOOH, and Co-NiS_v_@Co-NiOOH at zero potential (insert shows the molecular configuration of the elemental steps over Co-NiS_v_@Co-NiOOH)

Density functional theory (DFT) calculations were also performed to illustrate the high catalytic activity of Co, Cl-NiS. The aforementioned characterizations indicate the structure reconstruction of pristine nickel sulfide-based catalysts into nickel sulfide@oxyhydroxide. To illustrate the effect of Co and Cl co-doping on the intrinsic activity of catalyst, Ni_3_S_2_@NiOOH (abbreviated as NiS@NiOOH), Co-doped Ni_3_S_2_@Co-doped NiOOH (Co-NiS@Co-NiOOH), and Co-doped defective Ni_3_S_2_@Co-doped NiOOH (Co-NiS_V_@Co-NiOOH) models were constructed to represent the real electroactive phases of NiS, Co-NiS, and Co, Cl-NiS (Fig. S15), respectively. Figure 5a–c shows the impact of dopants on the electronic properties of the NiS@NiOOH derived from the bare NiS catalyst. The presence of the Co atom in the Co-NiS@Co-NiOOH and Co-NiS_V_@Co-NiOOH models leads to increased charge of the nearest Ni atoms, which should be due to the lower electronegativity of Co than Ni. Since the Cl and S etching-induced vacancies are in the inner Ni_3_S_2_ lattice, the impact of vacancy on the electronic property of surrounding Ni atoms is weak. Nevertheless, the calculated density of states (DOS) of the three systems imply that the Co-NiS_V_@Co-NiOOH model has an obviously higher *d*-band centre of -2.43 eV than Co-NiS@Co-NiOOH (− 2.62 eV) and NiS@NiOOH (− 2.90 eV) (Fig. 5d). Thus, the Co-NiS_V_@Co-NiOOH would have more empty antibonding states for the adsorption of reaction intermediates and improve the reaction process [11, 72]. Furthermore, the EGOR free-energy profiles were computed, and the results are shown in Fig. 5e. It is clear that all elemental steps show a down-hill feature in the EG-to-formate conversion process, suggesting that the EGOR process is thermodynamically favourable over the three catalyst surface. For NiS@NiOOH, the first step (formation of *glycolaldehyde) is the potential-determining step (PDS) with a high ΔG_*glycolaldehyde_ of -0.95 eV [73, 74], and the conversion of *glyoxal to *formate is an easier step. Differently, the introduction of Co dopants in Co-NiS@Co-NiOOH and Co-NiS_V_@Co-NiOOH shifts the PDS to the second step, namely the formation of *glyoxal. Compared to Co-NiS@Co-NiOOH, Co-NiS_V_@Co-NiOOH releases more energy at the PDS (-1.15 eV vs. -1.06 eV), indicating the conversion of *glycolaldehyde to *glyoxal over Co-NiS_V_@Co-NiOOH are more favourable than Co-NiS@Co-NiOOH [75]. These findings are in line with the experimental improved EGOR performance of Co, Cl-NiS. Hence, both Co dopant and the Cl dopant-induced vacancy contribute to the excellent catalytic performance of the self-evolved Co-NiS_V_@Co-NiOOH.

### Co, Cl-NiS Driven Real Plastic Electro-Upcycling

**Fig. 6:**
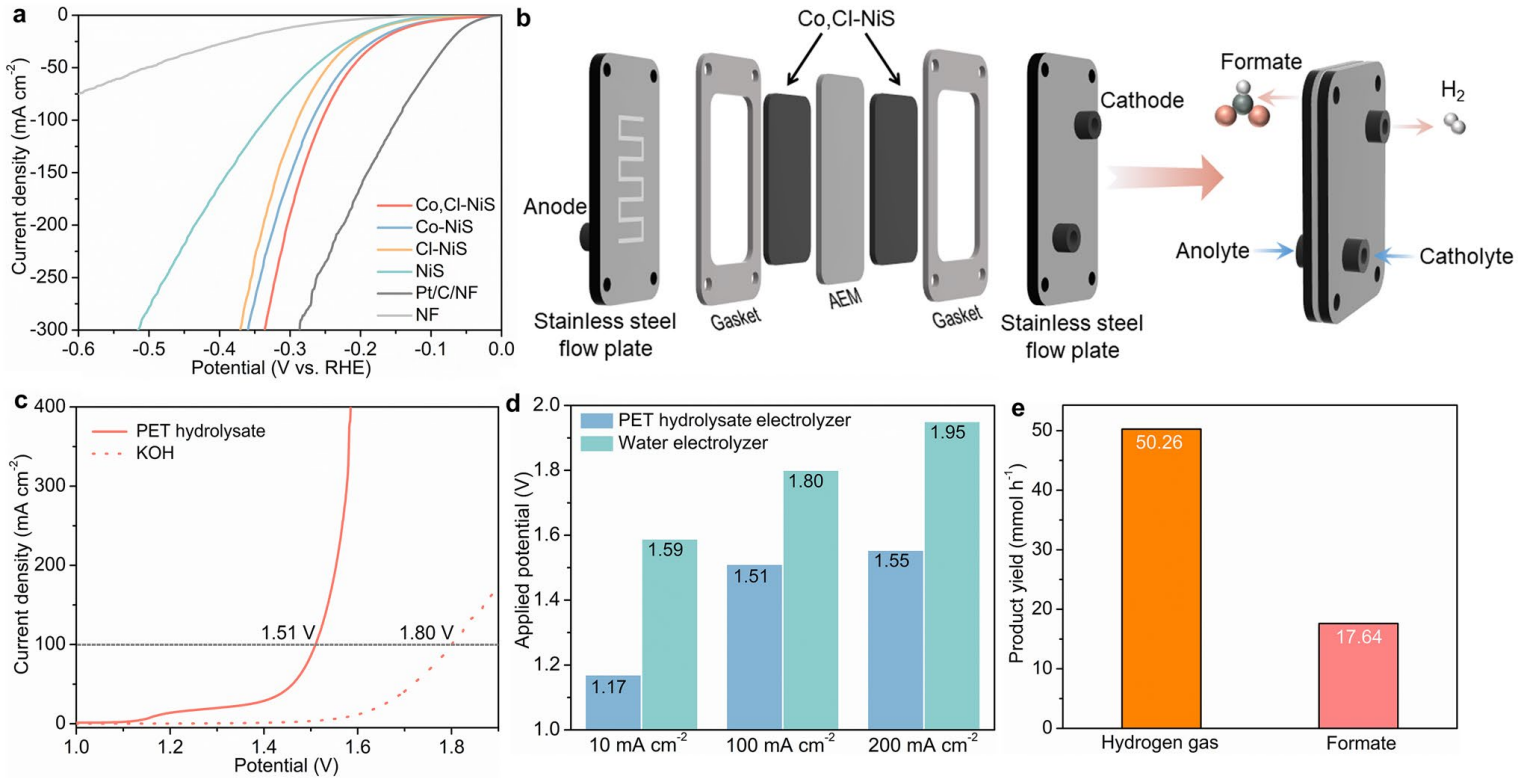
a LSV curves for HER of catalysts. **b** The MEA setup for PET hydrolysate electrolysis. **c** LSV curves of PET hydrolysate electrolysis and conventional water electrolysis (2 M KOH). **d** Applied potentials at different current densities for PET hydrolysate and alkaline water electrolysis systems. **e** Hydrogen production rate and anode product yield for PET hydrolysate electrolysis system

Considering the good EGOR performance of Co, Cl-NiS, a further attempt was conducted to demonstrate the energy-saving hydrogen production by real plastics electro-upcycling. First, the HER performance of catalysts was tested, and the LSV curves are depicted in Fig. 6a. Surprisingly, Co, Cl-NiS shows a good HER activity, with *η*_10_ = 98 mV. Although inferior to the Pt/C catalyst, Co, Cl-NiS outperforms the single-doped and undoped nickel sulfides (Fig. S16), as well as state-of-the-art HER electrocatalysts for alkaline HER (Table S4). In addition, the HER kinetics is analyzed by Tafel plots, Co, Cl-NiS shows a similar Tafel slope (62 mV dec^−1^) to the Pt/C catalyst (58 mV dec^−1^) (Fig. S17), suggesting the favourable HER kinetics of Co, Cl-NiS. The stability of Co, Cl-NiS for HER was also tested via recording the chronoamperometric curve for 24 h. Co, Cl-NiS can keep the high current density for the stability test, with only 0.6% activity loss (Fig. S18). Overall, Co, Cl-NiS is a high-performance bifunctional catalyst for EGOR and HER, which holds great promise for the real plastic electrolysis.

Initially, the PET waste (plastic water bottles, Fig. S19) was hydrolyzed in KOH solution at 80 °C, and the PET hydrolysate was used as the electrolyte. As suggested by previous studies, the EG monomer of PET can be selectively oxidized into formate while the TPA monomer will maintain in the solution [25]. The PET hydrolysate electrolysis was conducted in a home-made MEA flow reactor, as illustrated in Fig. 6b and S20. The bifunctional Co, Cl-NiS-assisted PET hydrolysate electrolysis only takes low potentials of 1.17, 1.51, and 1.55 V to achieve 10, 100 and 200 mA cm^−2^ respectively, which are 419, 290, and 397 mV lower than those of the conventional water electrolysis in 2 M KOH (Fig. 6c, d). The hydrogen production rate was further compared at a given potential of 1.7 V. The PET hydrolysate electrolyzer can produce hydrogen gas at an average rate of 50.26 mmol h^−1^ (Fig. 6e). In addition, the anode product yields were also calculated, and the PET hydrolysate electrolyzer can generate ~ 17.6 mmol formate per hour while the water electrolyzer can generate 0.46 mmol oxygen gas in the same period. Moreover, the PET electrolyte was treated with H_2_SO_4_ solution after electrolysis to regenerate white-coloured pure TPA (Fig. S21). Overall, the PET hydrolysate electrolyzer can not only realize energy-saving hydrogen production compared to conventional water electrolyzer but also produce value-added chemicals (formate and TPA) and contribute to the closed-loop utilization of plastic wastes.

## Conclusions

In summary, we demonstrate a dual-doping strategy to engineer high-performance Co, Cl-NiS electrocatalyst for PET upcycling. The hydrothermally synthesized Co, Cl-NiS shows an interconnected ultrathin nanosheet architecture, and the presence of Co and Cl dopants regulates the electronic structure of catalyst by upshifting the *d* band centre and facilitates the in situ structure reconstruction during the EGOR process. Compared with single doped and bare NiS, Co, Cl-NiS shows a better activity towards EGOR and only acquires 1.34 V versus RHE at a high current density of 100 mA cm^−2^. Also, Co, Cl-NiS can realize high efficiency and selectivity (> 90%) for EG-to-formate conversion at high current densities. At 1.7 V, the bifunctional Co, Cl-NiS-assisted real PET waste hydrolysate electrolysis can achieve a high H_2_ production rate (50.26 mmol h^−1^). This work paves the way for developing highly efficient bifunctional catalysts for plastic waste electro-upcycling, and would guide the development of advanced techniques for sustainable hydrogen production.

### Supplementary Information

Below is the link to the electronic supplementary material.

Supplementary file1 (PDF 1431 kb)
