# Supplementary material for: Dual-Doped Nickel Sulfide for Electro-Upgrading Polyethylene Terephthalate into Valuable Chemicals and Hydrogen Fuel
Source: Nanomicro Lett. 2023 Sep 11;15:210. doi: 10.1007/s40820-023-01181-8 (PMC10495299; doi:10.1007/s40820-023-01181-8)
Supplement: Supplementary file 1 — Supplementary file1 (PDF 1431 kb) [file 40820_2023_1181_MOESM1_ESM.pdf]

Supporting Information for

Dual-Doped Nickel Sulfide for Electro-Upgrading Polyethylene Terephthalate into Valuable Chemicals and Hydrogen Fuel

Zhijie Chen[1], Renji Zheng[2,] *, Teng Bao[3], Tianyi Ma[4], Wei Wei[1], Yansong Shen[5], Bing-Jie Ni[1,] *

[1] Centre for Technology in Water and Wastewater, School of Civil and Environmental Engineering, University of Technology Sydney, Sydney, NSW 2007, Australia

[2] School of Minerals Processing and Bioengineering, Central South University, Changsha 410083, P. R. China

[3] School of Biology, Food and Environment Engineering, Hefei University, Hefei 230601, P. R. China

[4] School of Science, STEM College, RMIT University, Melbourne, VIC 3000, Australia

[5] School of Chemical Engineering, University of New South Wales, Sydney, NSW 2052, Australia

*Corresponding authors. E-mail: zhengrj@csu.edu.cn (Renji Zheng); bingjieni@gmail.com (Bing-Jie Ni)

S1 Experimental

S1.1 Chemicals and Materials

Cobalt nitrate hexahydrate ($Co(NO_3)_2 \cdot 6H_2O$, 99.9%), sodium chloride (NaCl, 99.9%), thiourea (CH_4N_2S, 99.9%), ethanol (C_2H_6O, 99.8%), ethylene glycol ($C_2H_6O_2$, 99.8%), potassium hydroxide (KOH, 99.9%), concentrated hydrochloric acid (HCl, 37%), and concentrated sulfuric acid (H_2SO_4, 99.9%) were purchased from Fisher Scientific. 2-mm-thick nickel foam (NF) was bought from Phychemi Hong Kong Company Limited. The 5% Nafion 117 solution, commercial 20% Pt/C and RuO_2 (99.9%) catalysts were provided by Sigma-Aldrich Corporation. The anion exchange membrane (Sustainion X37-50) was obtained from Dioxide Materials. All the reagents were employed as obtained without further purification and distilled (DI) water (18.25 MΩ·cm) was utilized throughout all experiments was from a Millipore system. The polyethylene terephthalate plastic bottles were collected from the campus rubbish bins at University of Technology Sydney, Ultimo, Sydney, Australia.

S1.2 Pre-treatment of Nickel Foam

In this study, 2-mm-thick nickel foams (NF) was first cut into a rectangular shape. Then, the NF was ultrasonication sequentially in 3 M HCl, ethanol and DI water for 15 min respectively, with an aim to remove native oxides and other impurities on the surface.

After that, NF was rinsed three times with DI water and used for further hydrothermal treatment.

S1.3 Material Characterizations

The crystal structure of materials was determined by X-ray diffraction (XRD) measurements on a Rigaku Smart-Lab 9 kW diffractometer with the X-ray tube operated at 45 kV and 20 mA. The morphology of catalysts was recorded with scanning electron microscopy (SEM, Zeiss Sigma 500). The Raman spectra were tested on a ThermoFisher DXRi Raman microscope. Transmission electron microscopy (TEM) and high-resolution TEM (HRTEM) images were obtained on a TEM instrument (FEI Tecnai G2 F20 S-TWIN) with an acceleration voltage of 200 kV. Energy dispersive spectroscopy (EDS) mapping measurements were conducted by a TEM which is equipped with an EDS. The element composition and surface chemistry of catalysts were tested by the X-ray photoelectron spectroscopy (XPS, Thermo K-Alpha$^+$, Thermo Fisher Scientific, USA) with the Al (K_α) radiation. The ion concentrations in the electrolyte and element contents in catalysts were measured by an inductively coupled plasma mass spectrometry (ICP-MS) instrument (Agilent 7700s). ^{1}H and ^{13}C nuclear magnetic resonance (NMR) spectroscopy was recorded on a 600 MHz/AVANCE 400 (Bruker).

S1.4 Preparation of Pt/C and RuO_2 Electrodes

For the preparation of Pt/C and RuO_2 electrodes, 5 mg of commercial catalyst powder was first dispersed in 1 mL of mixed solution (500 μL of water, 450 μL of ethanol, and 50 μL of 5 wt% Nafion solution). After sonication for about 30 min, a homogeneous ink was obtained. Then, 100 μL of the ink was deposited onto a piece of acid-treated nickel foam (NF). After drying naturally, the electrode was obtained.

S1.5 Electrochemical Tests

The oxygen evolution reaction (OER), ethylene glycol oxidation reaction (EGOR), and hydrogen evolution reaction (HER) performance of catalysts was tested in a H-type cell, with a CHI 660E electrochemical workstation. The as-prepared NF-supported nickel sulfides were directly used as the working electrode. Hg/HgO serves as the reference electrode, and a graphite rod was the counter electrode. Linear sweep voltammetry (LSV) curves were recorded at a scan rate of 5 mV s^{-1}. The polarization curves were calibrated with 90% iR compensation to eliminate the solution resistance. To eliminate the contributions of oxidation current densities of metal species to EGOR and OER, LSV curves were recorded by sweeping from high to low potentials. All potentials measured were converted to the reversible hydrogen electrode (RHE) via the following equation: $E_{vs.\ RHE} = E_{vs.\ Hg/HgO} + 0.059\ pH + 0.098\ V$. For the EGOR tests, electrochemical impedance spectroscopy (EIS) was recorded at 1.32 V vs. RHE over the frequency range of 10^{-2} to 10^5 Hz, with an AC signal amplitude of 5 mV. The double-layer capacitances (C_{dl}) were calculated by conducting CVs at different scan rates (i.e., 10, 20, 30, 40, and 50 mV s^{-1}). Furthermore, the value of ECSA was calculated by the equation: $ECSA = S \times C_{dl} / C_s$, where C_{dl} is the double layer capacitance; C_s is the general

specific capacitance, the value is about 0.04 mF cm^{-2}, and S is the area of the working electrode. To test the long-term stability of catalysts, the chronoamperometric i-t curves were measured for 24 h.

To determine the Faradic efficiency (FE) and yield of formate at different potentials, the EGOR was carried out at the potentiostatic mode. 0.1 M EG in 1 M KOH was used as the electrolyte. After reactions, the concentration of substrate and products were analyzed by a nuclear magnetic resonance (NMR) spectrometer. ^{1}H and ^{13}C NMR spectra were recorded on a 600 MHz/AVANCE 400 (Bruker), In which 300 μL electrolyte was added with 300 μL D_2O and 30 μL dimethyl sulfoxide (DMSO) used as an internal standard. The Faradaic efficiency (FE), formate selectivity and yield were calculated with the following equations [S1, S2]:

$$\text{FE (\%)} = 100\% \times \frac{\text{N(formate)}}{\text{Total charge passed}/(\text{n} \times 96485)}$$

$$\text{Formate selectivity (\%)} = 100\% \times \frac{\text{N(formate)}}{\text{2N(converted EG)}}$$

$$\text{Formate yield (mmol cm}^{-2}\text{ h}^{-1}) = \frac{\text{N(formate)}}{\text{Electrode area} \times \text{reaction time}}$$

$$\text{Carbon balance (\%)} = 100\% \times \frac{\text{N(glycolate)} + \text{N(formate)}/2}{\text{N(consumed EG)}}$$

where N represents the moles of substances, n is the number of electron transfer for each product formation.

S1.6 Computational Methods

The calculations were performed using density functional density (DFT) implemented in the CASTEP package. The electron exchange-correlation energy was treated by the generalized gradient approximation (GGA) with Perdew-Burke-Ernzerhof (PBE) functional [S3]. Given the strong correlation effect of transition metals, DFT + U method was employed with the value of U_{eff} = 3.52 and 3.00 eV for Co and Ni atoms based on previous studies, respectively [S4]. γ-NiOOH is a layered structure with an interlayer of about 7 Å. Nevertheless, the definite structure of metal ions and water molecules between the NiO_2 layers has not been well characterized. To avoid uncertainty caused by the interlayer species, the structure was approximated by shortening the interlayer distance and ignoring the interlayer species. The simplified γ-NiOOH has a calculated lattice constant of $a = b = 0.295$ nm and $c = 1.307$ nm. This model captures the layered NiO_2 structure and local bonding environment of Ni. Similar approximation approaches can be found in previous studies [S5-S7]. The Ni_3S_2@NiOOH heterostructure was constructed to represent the real active phase of bare NiS catalyst due to structure reconstruction. Similarly, Co-doped Ni_3S_2@Co-doped NiOOH (Co-NiS@Co-NiOOH), and Co-doped defective Ni_3S_2@Co-doped NiOOH (Co-NiS_V@Co-NiOOH) models were constructed to represent the real electroactive phases of Co-NiS and Co,Cl-NiS, respectively. A 3 × 3 × 1 k-mesh was

employed for the Brillouin zone integrations, and a vacuum layer of 20 Å was used to avoid possible interactions between the images. The kinetic energy cutoff was set to 500 eV for the plane-wave basis set. For heterojunction surface calculations, Brillouin zone integration was sampled with the 2 × 2 × 1 Monkhorst-Pack mesh k-point. During the geometry optimizations step, the bottom pyrite and ilmenite slabs were constrained at the bulk position and the surface slab was relaxed. The EGOR process was computed based on the experimental results in Figs. 3 and S10-S12, and previous reports [S8].

S2 Supplementary Figures and Tables

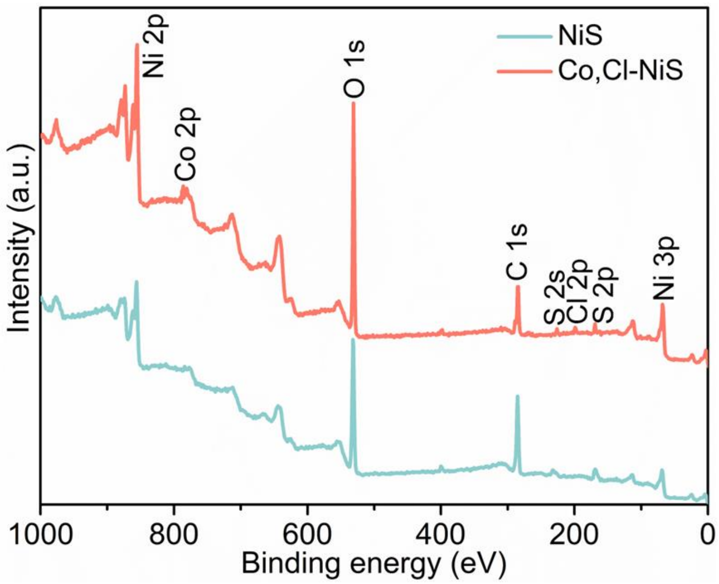

Fig. S1 XPS survey spectrum of Co,Cl-NiS and NiS

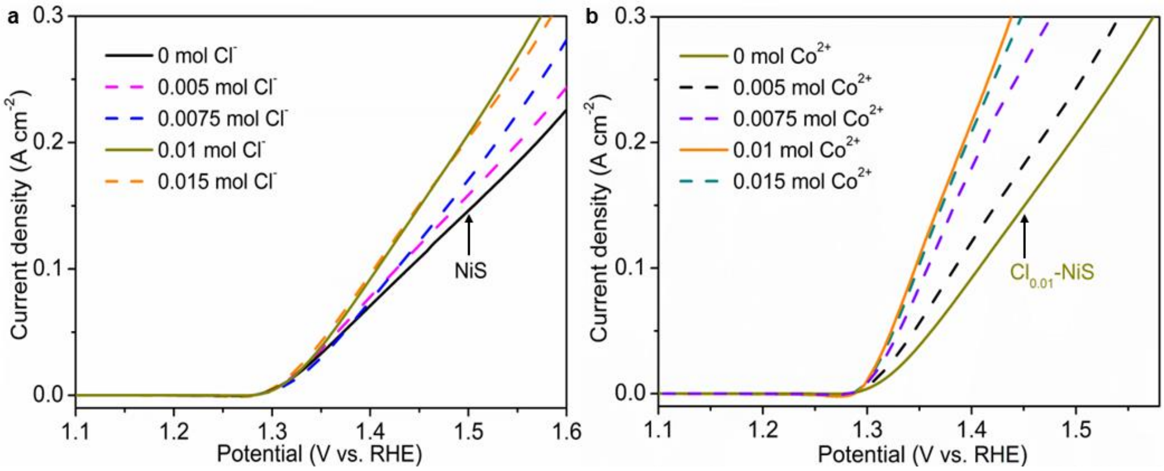

Fig. S2 (**a**) Effect of Cl doping level on the catalytic performance of NiS. (**b**) Effect of Co doping level on the catalytic performance of $Cl_{0.01}$-NiS ($Cl_{0.01}$ means the dosage of Cl precursor is 0.01 mol)

To determine the optimal doping levels of Cl and Co, a series of NiS-based materials were synthesized. Firstly, different amounts of Cl^- were added into the reaction solution

to synthesize a series of Cl-doped NiS. and the catalyst prepared with 0.01 mol Cl^- shows the best catalytic activity (Fig. S2a). Then, the doping level of Co^{2+} was investigated by adding different amounts of Co^{2+} into the reaction solution, with the presence of 0.01 mol Cl^-. **Figure** S2b indicates that the utilization of 0.01 mol Co^{2+} and 0.01 mol Cl^- precursors leads to the optimal catalytic performance.

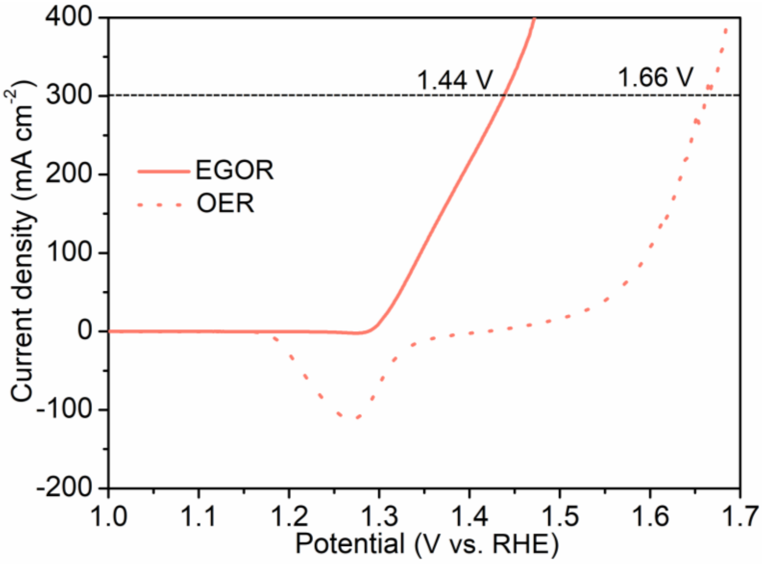

Fig. S3 OER and EGOR LSV curves of Co,Cl-NiS

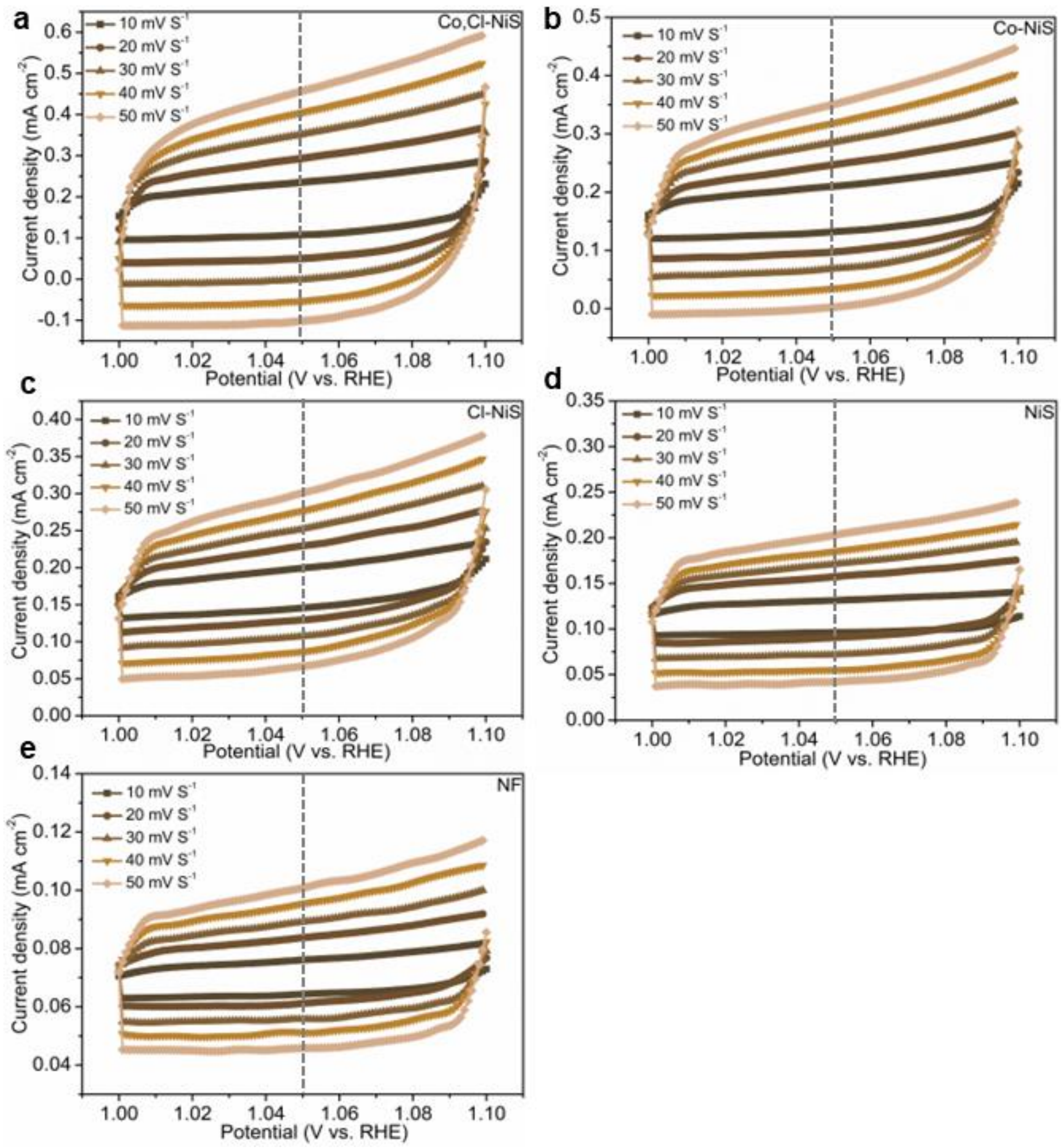

Fig. S4 Cyclic voltammograms of (**a**) Co,Cl-NiS, (**b**) Co-NiS, (**c**) Cl-NiS, (**d**) NiS and (**e**) NF at different scan rates (from 10 to 50 mV s^{-1} with an increment of 10 mV s^{-1})

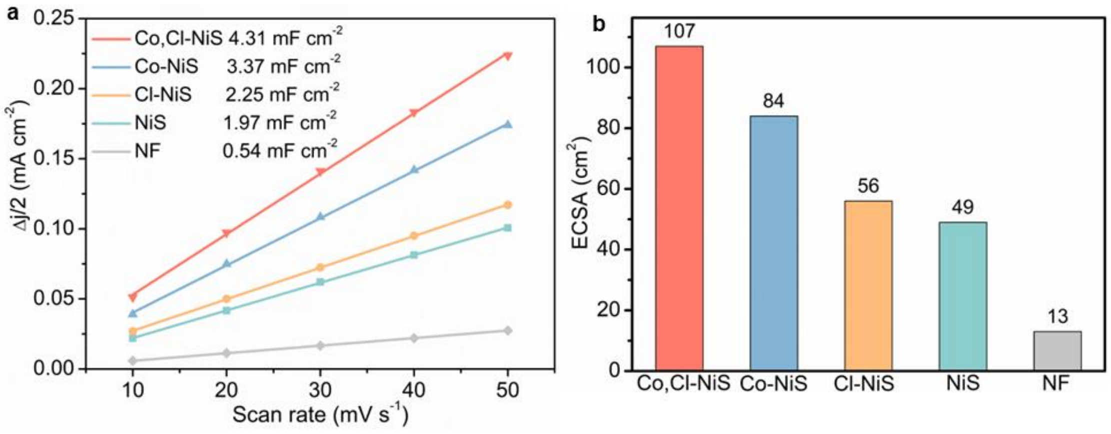

Fig. S5 (**a**) The difference in current density ($\Delta j = (j\text{a} - j\text{c})/2$) plots against the scan rate of Co,Cl-NiS, Co-NiS, Cl-NiS, NiS and NF. (b) ECSA values of catalysts

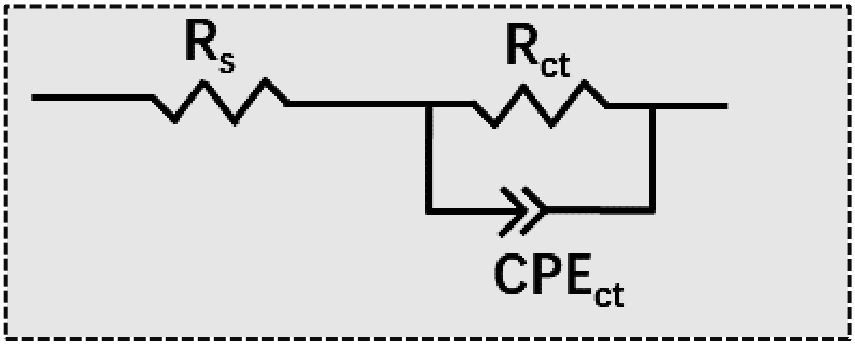

Fig. S6 Equivalent circuit model for EIS data fitting

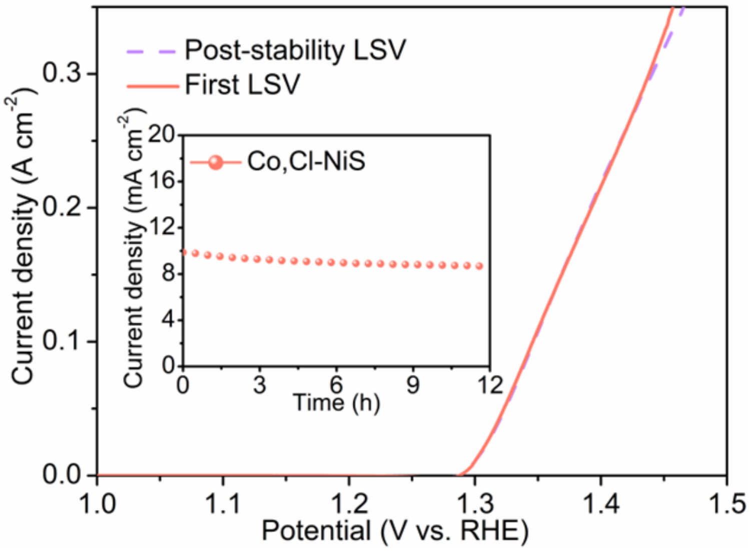

Fig. S7 LSV curves of Co,Cl-NiS before and after 12 h EGOR test. (The post-stability LSV was tested in a pristine 1M KOH + 0.1 M EG solution). Insert shows the chronoamperometric curve of Co,Cl-NiS for the 12 h EGOR test.

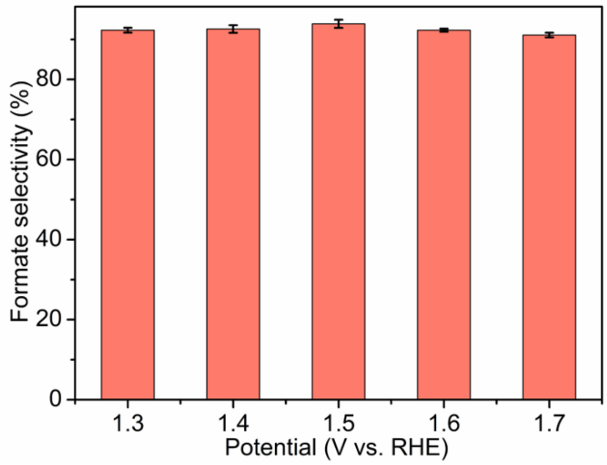

Fig. S8 Formate selectivity of Co,Cl-NiS at varied applied voltages

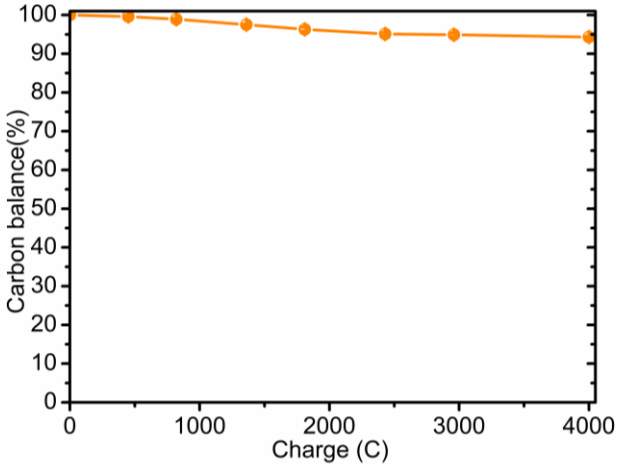

Fig. S9. Carbon balance of EGOR as a function of input charge

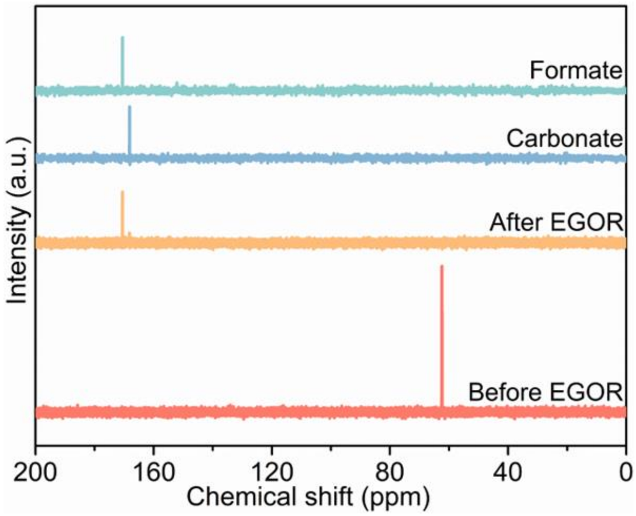

Fig. S10 ^{13}C NMR spectra of liquid products before and after EGOR process

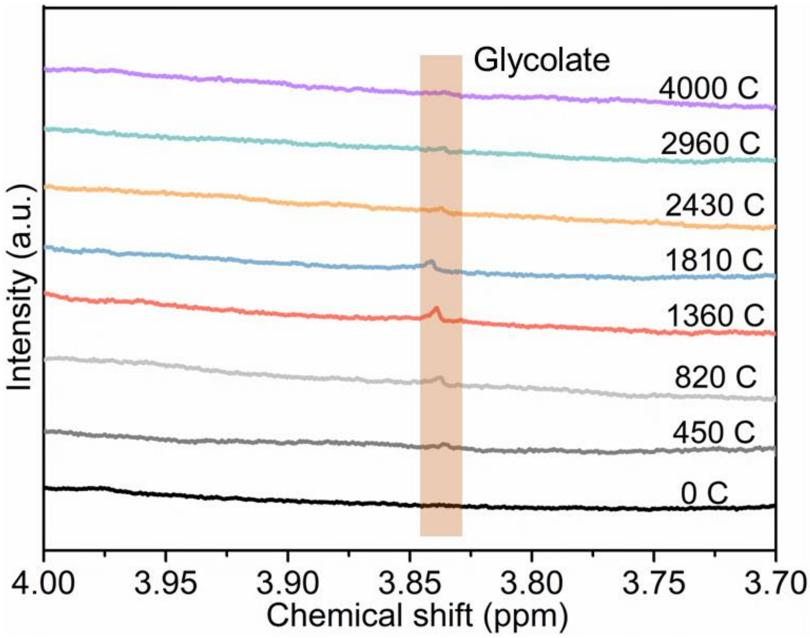

Fig. S11 An enlarged part of the ^{1}H NMR spectra of products during EGOR process

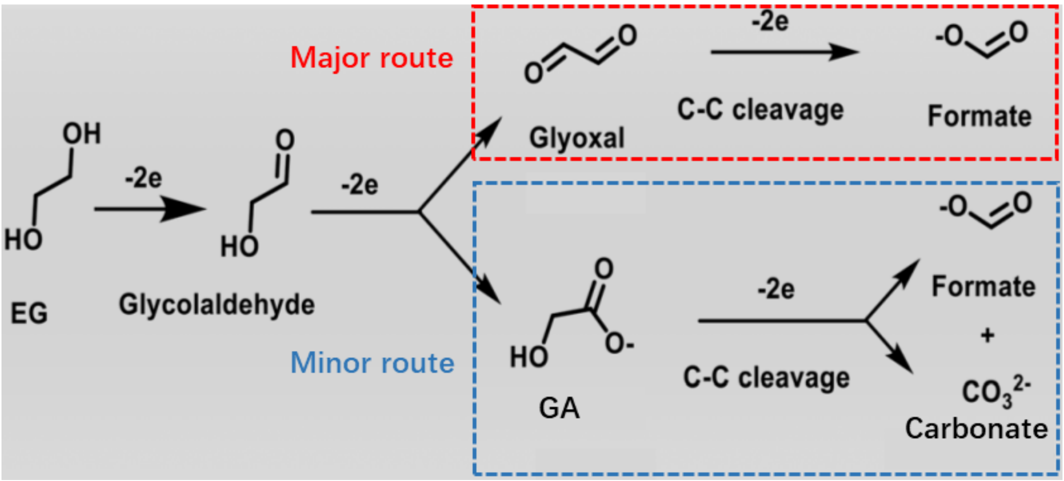

Fig. S12 Proposed possible EGOR mechanism over the Co,Cl-NiS catalyst under alkaline conditions

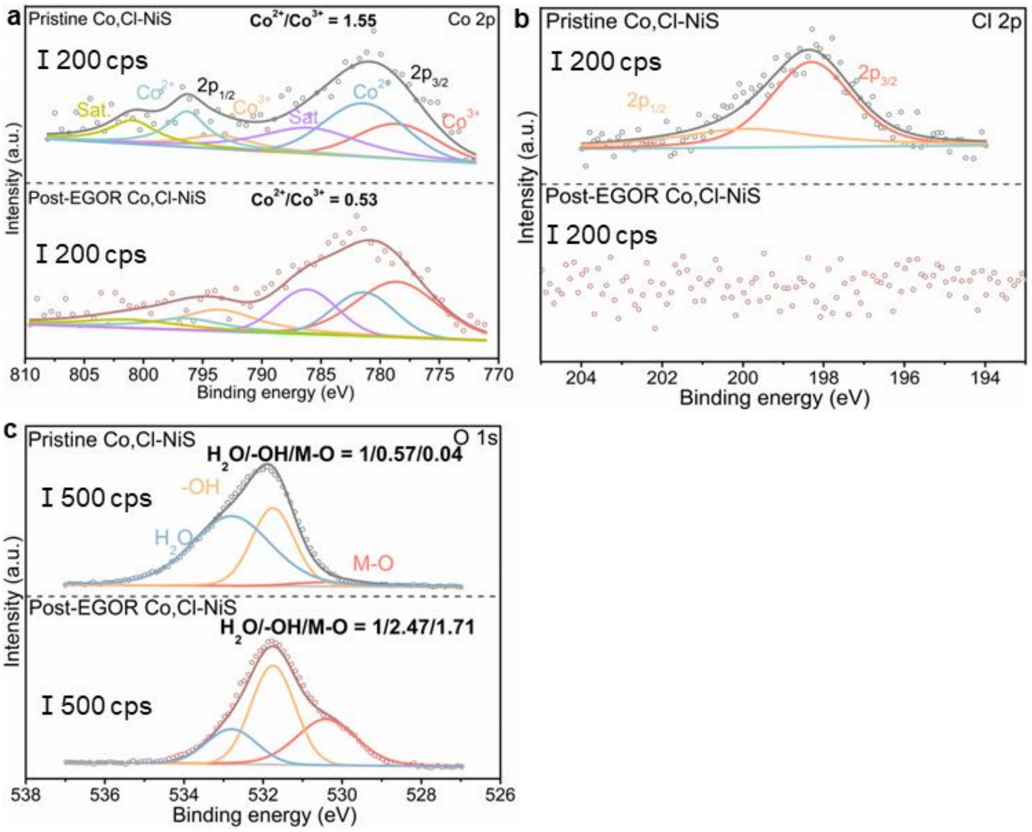

Fig. S13 High-resolution XPS spectra for (**a**) Co 2p, (**b**) Cl 2p, and (**c**) O 1s of Co,Cl-NiS before and after EGOR process

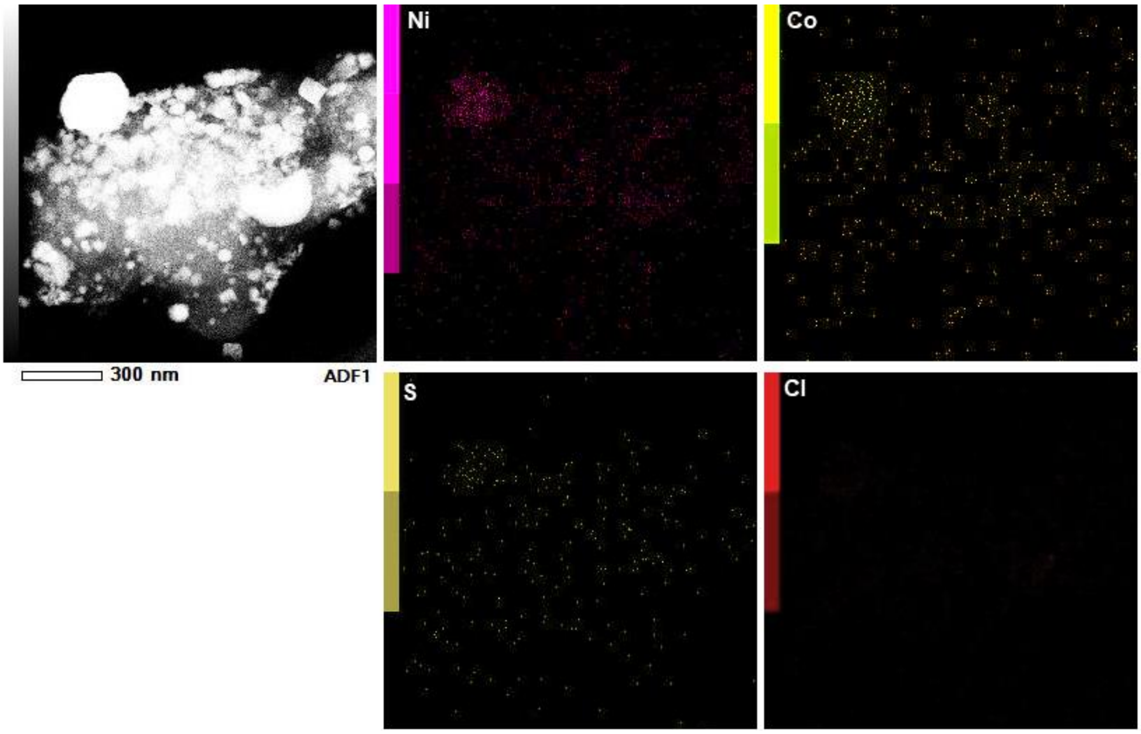

Fig. S14 HAADF-STEM and corresponding elemental mapping images of post-EGOR Co,Cl-NiS

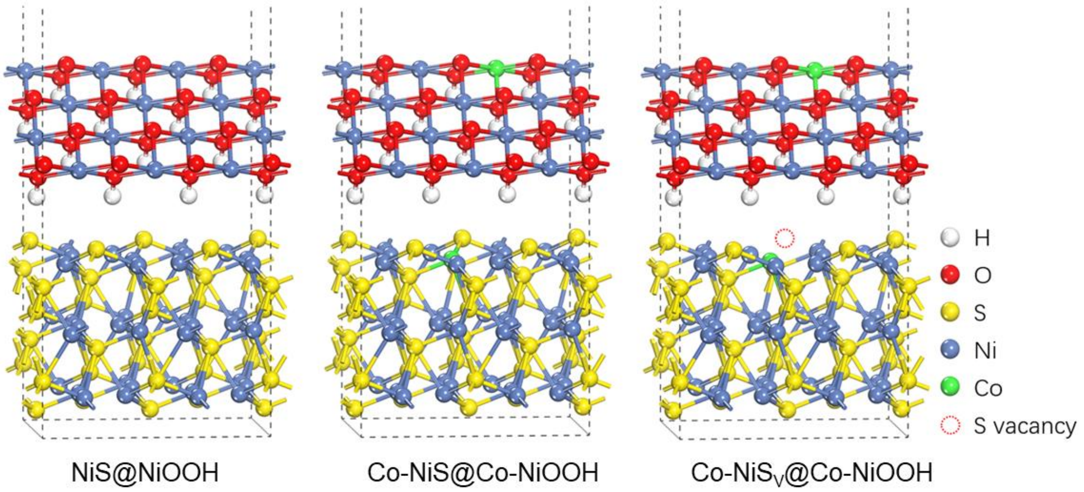

Fig. S15 Constructed NiS@NiOOH, Co-NiS@Co-NiOOH, and Co-NiS$_v$@Co-NiOOH models for DFT calculations. Since Cl is a dopant and replace some S sites in the Ni_3S_2 crystal structure, the electrochemical etching of Cl will thus lead to S vacancy

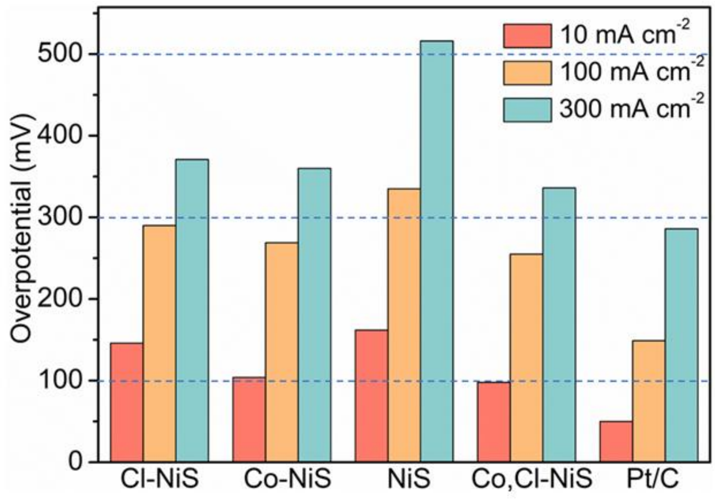

Fig. S16 Comparison of HER overpotentials of catalysts at different current densities

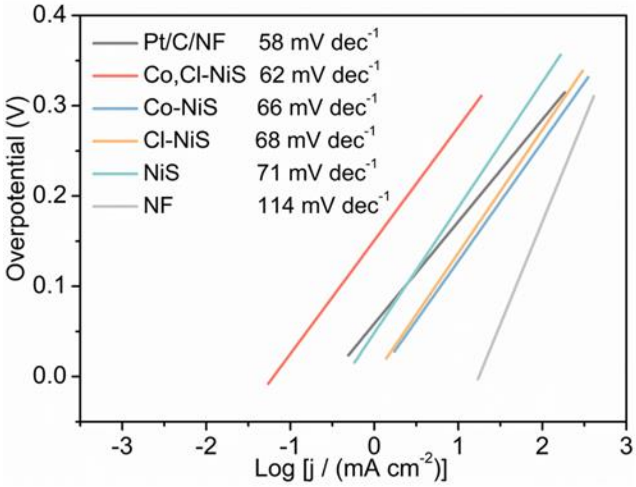

Fig. S17 Tafel plots of catalysts for HER

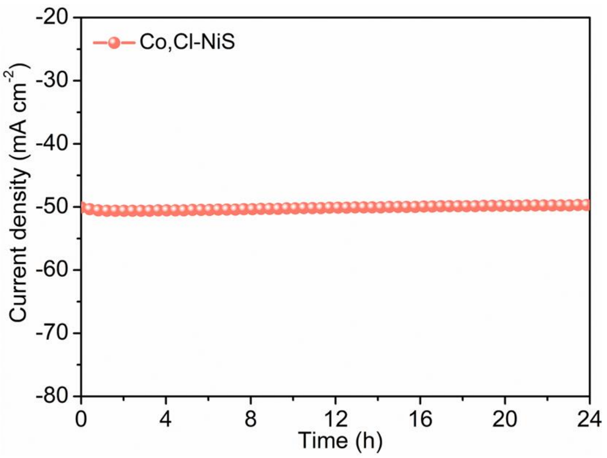

Fig. S18 Chronoamperometric curve of Co,Cl-NiS for the 24 h HER test

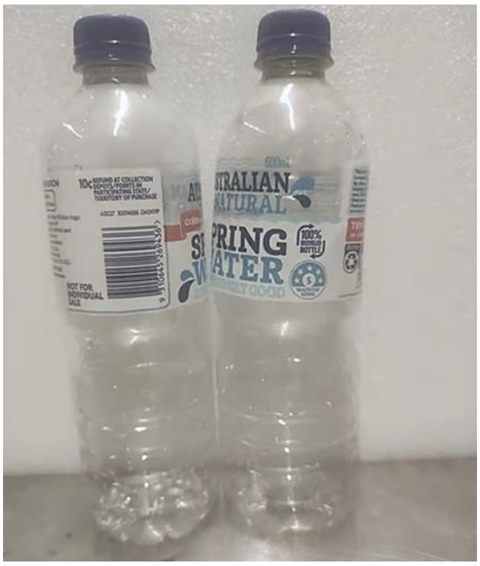

Fig. S19 Picture of the collected PET water bottles

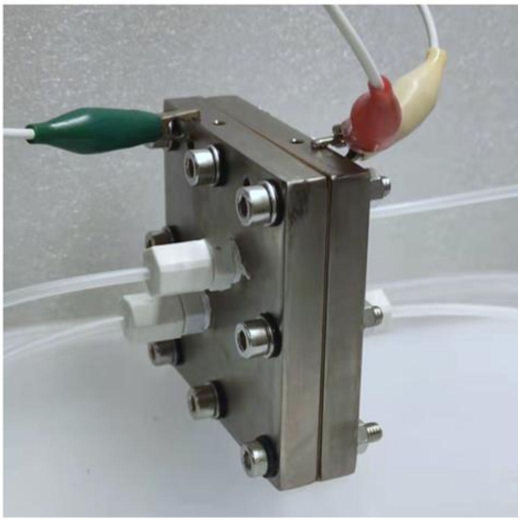

Fig. S20 Picture of the home-made membrane-electrode assembly (MEA) flow electrolyzer

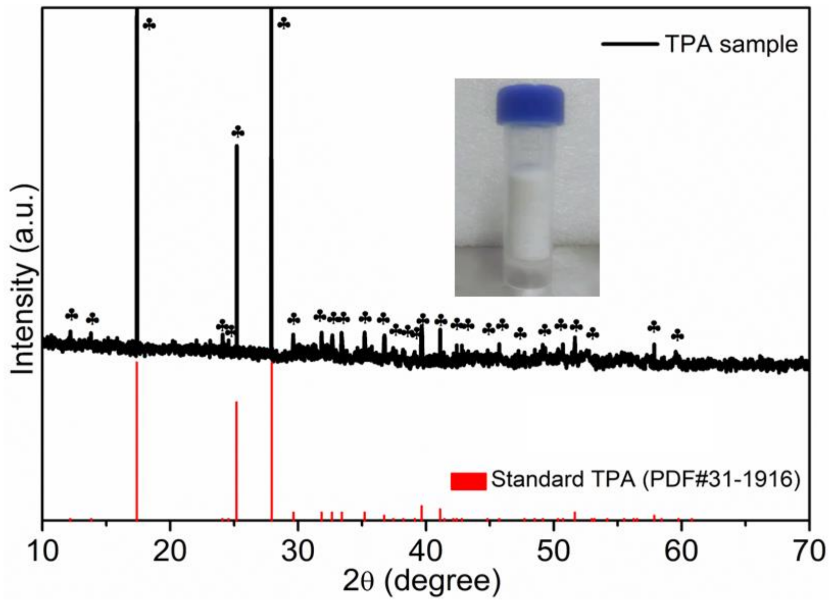

Fig. S21 XRD pattern of the recovered TPA, and the insert shows a picture of the recovered TPA

Table S1 A summary of OER properties of Co,Cl-NiS and state-of-the-art electrocatalysts in 1 M KOH

Catalyst	η_{10} (mV)	References
CeO_2-$CoS_{1.97}$	264	*Adv. Mater. 2021, 2102593.*
$NiMoO_4$ NRs-Fe-1	351	*J. Am. Chem. Soc. 2021, 143, 14169-14177.*
NiAs/GC	360	*Adv. Energy Mater. 2019, 1900796*
Fe-$NiSe_2$/GC	268	*Angew. Chem. Int. Ed. 2018, 57, 4020-4024.*
$NiCeO_x$/NF	295	*ACS Catal. 2019, 92, 1605-1611.*
$NH_4CoPO_4{\cdot}H_2O$	252	*Adv. Sci. 2021, 2100498.*
FeAs/NF	252	*Chem. Sci., 2020, 11, 11834.*
Fe-doped Co_3O_4	262	*Adv. Mater. 2020, 2002235.*
FeCoNiMo high-entropy alloy	250	*ACS Catal. 2022, 12, 17, 10808–10817.*
Co-doped $Fe_5(PO_4)_4(OH)_3{\cdot}H_2O$	290	*Appl. Catal. B 2022, 300, 120712.*
MoS_2/NiS_2 NSs	278	*Adv. Sci. 2019, 6, 1900246.*
Ti-CoS_x	249	*Small. 2022, 18(4), 2103106.*
$La_{0.9}Ce_{0.1}NiO_3$	270	*Adv. Energy Mater. 2021, 11, 2003755.*
$Ba_5Bi_{0.25}Co_{3.75}FeO_{14-\delta}$	308	*Adv. Mater. 2021, 2103392.*
Ni/$NiFeMoO_x$/NF	255	*Adv. Sci. 2020, 7, 1902034.*
Ni–Fe–Se nanocages	249	*Adv. Mater. 2021, 2103004.*
**Co,Cl-NiS**	**241**	***This study***

Table S2 A summary of EGOR properties of Co,Cl-NiS and state-of-the-art noble metal-free electrocatalysts

Catalyst	Current density ($mA\ cm^{-2}$)	Potential (V vs. RHE)	Electrolyte	References
Ni_3N/W_5N_4	10	1.33	PET hydrolysate	*Appl. Catal. B 2022, 307, 121198.*
$NiCo_2O_4$/CFP	50	1.44	1 M NaOH + 0.1 M EG	*ACS Catal. 2022, 12, 11, 6722–6728.*
OMS-Ni_1-CoP	50	1.38	1 M KOH + 0.5 M EG	*Appl. Catal. B 2022, 316, 121667.*
CuO	10	1.38	PET hydrolysate	*J. Phys. Chem. Lett. 2022, 13, 2, 622–627*
Co-Ni_2P/NF	150	1.42	1 M NaOH + 5 g/L PET	*Sustainable Energy Fuels 2022,6, 4916-4924.*
Cobalt-Based Coordination Polymer	10	1.42	1 M KOH + 0.1 M EG	*ACS Catal. 2023, 13, 1, 469–474.*
**Co,Cl-NiS**	**10**	**1.30**	1 M KOH + 0.1 M EG	**This study**
	**50**	**1.32**	-	-
	**100**	**1.34**	-	-
	**150**	**1.37**	-	-

Table S3 Calculated charge transfer resistance (R_{ct}) and solution resistance (R_s) (in Ohm, Ω) of catalysts obtained from the Nyquist plot during the EIS experiments

Catalyst	R_s	R_{ct}
Co,Cl-NiS	1.57	3.7
Co-NiS	1.58	6.1
Cl-NiS	1.61	8.4
NiS	1.63	19.5
NF	1.71	123

Table S4 A summary of HER properties of Co,Cl-NiS and state-of-the-art electrocatalysts

Catalyst	Electrolyte	η_{10} (mV)	References
Fe-doped $CoWO_4$	1.0 M KOH	118	*J. Mater. Chem. A 2021, 9, 9753-9760*
$SrCo_{0.85}Fe_{0.1}P_{0.05}O_{3-\delta}$ nanofilm	1.0 M KOH	110	*Adv. Mater. 2018, 30, 1804333.*
$Mn_3(PO_4)_2$	1.0 M KOH	104	*Appl. Catal. B. 2021, 292, 120202.*
Reduced $NiCo_2O_4$	1.0 M KOH	135	*J. Am. Chem. Soc. 2018, 140, 13644–13653.*
Mo_2NiB_2	1.0 M KOH	160	*Small. 2022, 18(6), 2104303.*
o-$CoSe_2$|P	1.0 M KOH	104	*Nat. Commun. 2018, 9, 2533.*
Fe-doped MOF CuCoSe@HCNF	1.0 M KOH	181	*Appl. Catal. B. 2021, 293, 120209.*
N-doped Ni_3S_2/NF	1.0 M KOH	155	*Adv. Energy Mater. 2018, 8, 1703538.*
Mn-CoP nanowire@Mn-CoOOH nanosheet	1.0 M KOH	110	*Appl. Catal. B. 2021, 292, 120172.*
Ni NP|Ni–N–C/EG	1.0 M KOH	147	*Energy Environ. Sci., 12 (2019) 149-156.*
N-$NiMoO_4$/NiS_2	1.0 M KOH	99	*Adv. Funct. Mater. 2019, 29, 1805298.*
1T-Fe/P-WS_2@CC	1.0 M KOH	116	*Appl. Catal. B. 2021, 286, 119897.*
Cu NDs/Ni_3S_2 NTs-CFs	1.0 M KOH	128	*J. Am. Chem. Soc. 2018, 140, 610–617.*
MoS_2/Co_9S_8/Ni_3S_2/Ni	1.0 M KOH	113	*J. Am. Chem. Soc. 2019, 141, 10417-10430.*
Ni_3S_2/VG@NiCo LDHs	1.0 M KOH	120	*Chem. Eng. J. 2021, 415, 129048.*
$NiSe_2$/Ni_3Se_4/NF	1.0 M KOH	145	*Appl. Catal. B 2022, 303, 120915*
**Co,Cl-NiS**	**1.0 M KOH**	**98**	***This study***

Supplementary References

[S1] M. Du, Y. Zhang, S. Kang, C. Xu, Y. Ma et al., Electrochemical production of glycolate fuelled by polyethylene terephthalate plastics with improved techno-economics. Small 2303693 (2023). https://doi.org/10.1002/smll.202303693

[S2] H. Zhou, Y. Ren, Z. Li, M. Xu, Y. Wang et al., Electrocatalytic upcycling of polyethylene terephthalate to commodity chemicals and H_2 fuel. Nat. Commun. **12**, 4679 (2021). https://doi.org/10.1038/s41467-021-25048-x

[S3] L. Gao, X. Cui, Z. Wang, C.D. Sewell, Z. Li et al., Operando unraveling photothermal-promoted dynamic active-sites generation in $NiFe_2O_4$ for markedly enhanced oxygen evolution, Proc. Natl. Acad. Sci. USA **118** 23421118 (2021). https://doi.org/10.1073/pnas.2023421118

[S4] X. Yu, C. Hu, P. Ji, Y. Ren, H. Zhao et al., Optically transparent ultrathin NiCo alloy oxide film: Precise oxygen vacancy modulation and control for enhanced electrocatalysis of water oxidation. Appl. Catalysis B: Environmental **310**, 121301 (2022). https://doi.org/10.1016/j.apcatb.2022.121301

[S5] M. Wang, B. Zhang, J. Ding, F. Zhang, R. Tu et al., A robust approach to in situ exsolve highly dispersed and stable electrocatalysts. Small **18**, 2105741(2022). https://doi.org/10.1002/smll.202105741

[S6] D. Friebel, M.W. Louie, M. Bajdich, K.E. Sanwald, Y. Cai et al., Identification of highly active Fe sites in (Ni, Fe) OOH for electrocatalytic water splitting. J. Am. Chem. Soc. **137**, 1305-1313 (2015). https://doi.org/10.1021/ja511559d

[S7] F. Song, M.M. Busch, B. Lassalle-Kaiser, C.-S. Hsu, E. Petkucheva et al., An unconventional iron nickel catalyst for the oxygen evolution reaction. ACS Centr. Sci. **5**, 558-568 (2019). https://doi.org/10.1021/acscentsci.9b00053

[S8] F. Liu, X. Gao, R. Shi, C. Edmund, Y. Chen, A general electrochemical strategy for upcycling polyester plastics into added-value chemicals by a $CuCo_2O_4$ catalyst. Green Chemistry **24**, 6571-6577 (2022). https://doi.org/10.1039/D2GC02049A
